# Insights into pig resilience: the Microbiome-genetic connection

**DOI:** 10.1186/s40813-026-00506-4

**Published:** 2026-03-30

**Authors:** Enrico Mancin, Cristina Casto-Rebollo, Christian Maltecca, Noelia Ibañez-Escriche, Roberto Mantovani, Cristina Sartori, Francesco Tiezzi

**Affiliations:** 1 Department of Agronomy, Food, Natural Resources, Animals and Environment-DAFNAE, University of Padova, 35020 Legnaro, Italy; 2 Institute for Animal Science and Technology, Universitat Politècnica de València, Valencia, 46022 Spain; 3 Department of Animal Science, North Carolina State University, Raleigh, NC 27695 USA; 4 Department of Agriculture, Food, Environment, and Forestry (DAGRI), University of Florence, 50144 Florence, Italy

**Keywords:** Resilience, Microbiota, Precision farming, Heat-Stress, welfare

## Abstract

Animal resilience and the gut microbiota have emerged as two major focus areas in animal science, particularly in swine production. The gut microbiota, as a dynamic and responsive ecosystem, offers unique potential to describe and interpret complex host phenotypes, reflecting genetic and environmental influences and their interplay. In parallel, enhancing animal resilience represents a key opportunity for swine production systems facing increasing pressures from climate change, sustainability requirements, and efficiency constraints, compounded by narrow profit margins and reduced labor availability per pig. Together, these factors necessitate pigs that can robustly cope with environmental and management challenges with minimal human intervention. These pressures threaten not only economic sustainability but also raise serious animal welfare concerns. However, improving resilience remains challenging because it (i) lacks a directly measurable trait and (ii) is difficult to enhance through environmental management, due to its inherent definition, and also through genetic selection, owing to its low to moderate heritability. In this context, the microbiota may capture a component of resilience-related variability that is not explained by host genetics or environmental factors alone. Accordingly, microbiota data, with their dual capacity to integrate host genetic background and environmental exposures, emerge as a valuable tool not only for monitoring resilience but also as a potential target for its improvement. In this review, we first define resilience in swine, outlining how it can be monitored and measured. We then discuss the importance of the microbiota, especially gut microbiota, in swine production. Finally, we explore the biological connections between resilience and the host microbiota, highlighting opportunities to leverage this relationship to address current and future challenges in swine systems.

## Background

The interplay between host resilience and the gut microbiota is increasingly recognized as a critical component of swine production, with important implications for breeding strategies, health management, and the long-term sustainability of production systems. This growing relevance is closely linked to the profound structural changes the swine industry has undergone since the 1960s, evolving into a highly industrialized, intensive, and globalized system characterized by increased specialization among countries [1]. These transformations have been largely driven by narrowing profit margins and increasing profit volatility, which have intensified pressure for higher production efficiency and promoted the widespread adoption of large-scale, intensive production systems [2]. In response to increasing economic pressures, many farms have made their production systems more efficient to maintain productivity and economic viability. However, this transition has introduced new challenges, particularly regarding animal health and welfare [3, 4]. Indeed, modern pig production systems are often large and complex, with high stocking densities and limited individualized care to minimize labor costs. These factors, combined with a less specialized workforce and limited monitoring capacity, make preserving animal welfare more difficult [5]. As a result, pigs have become more susceptible to a range of stressors, including endemic and epidemic diseases, as well as production-related issues such as mortality, post-weaning disorders, and locomotion problems [6, 7]. This increased susceptibility is also partly the result of long-term genetic selection emphasizing traits such as growth rate and feed efficiency, sometimes at the expense of robustness and resilience to stress or disease [7].

These challenges are further exacerbated by climate change, which imposes additional pressures on animal production systems [8]. Beyond direct thermal stress, increasing environmental variability also compromises animals’ ability to cope with fluctuations in feed availability and quality, rendering resilience to nutritional stress an increasingly important trait [9].

The combination of these previously discussed factors: (i) a more demanding production environment, (ii) a reduced functionality due to specialization, and (iii) emerging environmental challenges, underscores the need for breeding and management strategies that jointly support both performance and robustness. Addressing these combined pressures requires integrated adaptation approaches, including targeted breeding strategies, improvements in farm management practices, and the development of early warning systems. However, the effectiveness of such interventions is highly context dependent, varying according to local environmental conditions, production systems, and resource availability [10].

In this evolving context, scientific interest in the resilience of livestock species has grown substantially in response to these challenges [7]. Resilient animals, those capable of withstanding, adapting to, and recovering from disturbances, have become a central focus in discussions of sustainable livestock and swine production systems. This emphasis reflects both the operational realities of intensive production environments and the opportunity to exploit genetic variation that supports stable performance under variable or suboptimal conditions [11]. Despite this growing interest, selecting for and improving resilience remains challenging not only because of its complex and multifactorial nature, but also due to fundamental limitations in how the trait can be captured (e.g., recorded) and exploited within breeding programs. Resilience is not directly observable, is difficult to define in a generalizable manner, and currently lacks standardized metrics for routine measurement and quantification [12]. Even when resilience-related traits can be characterized [13], available indicators are often highly context-specific, reflecting resistance or tolerance to particular stressors rather than a general capacity to cope with diverse and unpredictable challenges [14]. Moreover, reported heritability estimates for resilience indicators range from near-zero to moderate (up to 0.49 [15, 16]), indicating that resilience is governed by multiple interacting genetic and environmental factors. As a result, even well-defined indicators may yield limited genetic gain, reducing the effectiveness of conventional selection strategies and emphasizing the need to incorporate complementary, more heritable sources of information or proxy traits to support genetic improvement in resilience.

In parallel with the growing emphasis on resilience, microbiome studies have gained substantial momentum in swine research [17]. The gut microbiota has contributed to a deeper understanding of a wide range of complex traits, including meat quality [18], feeding behavior [19], and growth performance [20], by reflecting underlying biological processes that connect host genetics with environmental and physiological responses. This contribution stems from the fact that microbial variation reflects a complex interplay between host genetics, environmental conditions, and internal physiological states such as age, health status, and exposure to stress. Consequently, the microbiota is increasingly recognized not only as a biomarker of the host’s physiological condition [21], but also as a promising tool for predicting and selecting complex phenotypes [22].

Therefore, the microbiota may help address several challenges associated with effective selection for resilience. One major limitation is the difficulty of defining and quantifying resilience in a broad, generalizable manner beyond specific diseases or isolated stress conditions. In this context, the microbiota, through its rapid and measurable responses to environmental perturbations, distinctive temporal dynamics, and host-specific signatures, may reflect an animal’s cumulative capacity to cope with and recover from diverse stressors, thereby serving as a biologically informative proxy for resilience [23]. In addition, the microbial community likely captures a component of phenotypic variance in resilience that is not fully explained by host genetics or measured macro-environmental factors alone. Indeed, emerging evidence indicates that microbial profiles provide additional insight into complex traits such as longevity and fertility [24], supporting their potential role in resilience-oriented breeding programs and in improving our understanding of pigs’ adaptive capacity [25]. Finally, beyond serving as a biomarker, the microbiota may function as a biological mediator of resilience through its involvement in immune, metabolic, and stress-response pathways. This raises the possibility of improving resilience by deliberately modulating microbial composition or activity via nutritional or management-based interventions. Taken together, these considerations highlight the microbiota as both an integrative indicator of resilience and a complementary source of phenotypic information for genetic improvement, while motivating a broader conceptual framework that views the host and its microbiota as a functional biological unit (the holobiont).

In light of this perspective, this review aims to: (i) examine the factors determining across- and within-pig phenotypic variation; (ii) define and evaluate current concepts and measures of resilience in pigs; (iii) outline the holobiont concept and describe the formation and progression of the pig microbiome, with particular emphasis on the gut microbiota; (iv) explore the potential interplay between resilience and the microbiota; and (v) discuss how microbiome-informed approaches could be integrated into future breeding and management strategies to enhance resilience in modern pig production system

## Factors influencing across- and within-pig phenotypic variation

Before addressing resilience and the role of the microbiome, it is important to first understand why individual animals differ in their phenotype. A pig’s phenotype, which is defined as its observable trait, such as growth rate, disease resistance, and resilience, arises from the complex interplay between its genetic makeup, environmental exposures, and random biological variation, in addition to all their interactions. Genetic differences provide the blueprint for potential performance, while environmental factors such as nutrition, housing, and health management modulate how this potential is expressed. Additionally, gene-environment interactions and epigenetic mechanisms further shape phenotypic outcomes, leading to considerable variability among animals in their ability to perform and adapt under stress.

### The classic view: genes and environment

**Fig. 1:**
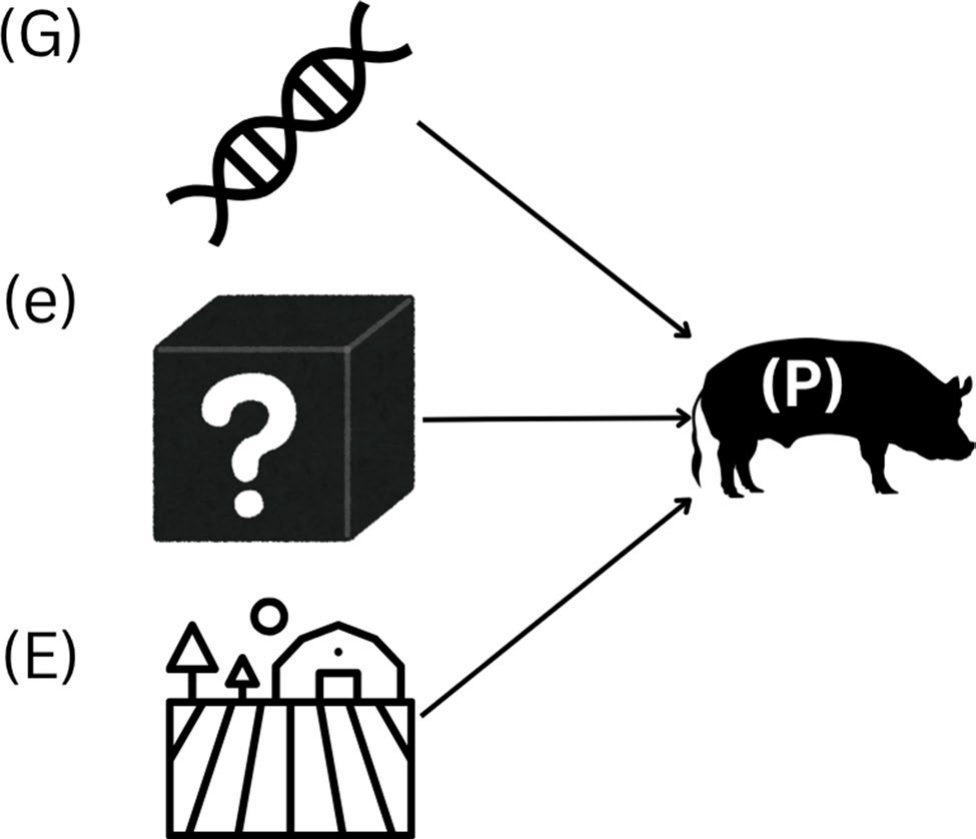
Conceptual model of phenotype (**P**) as the sum of genetic (**G**), environmental (**E**), and random, uncontrollable environmental effects (**e**)

Traditionally, the phenotype has been conceptualized as the sum of genetic and environmental influences, along with stochastic variation (Fig. 1). This can be expressed as:

$${\rm{P = G + E + e}}$$

where the term ‘G’ represents the genetic contribution, determined by the inherited genomic information that defines the biological potential of the pig. Genetic variation underlies differences in traits such as growth efficiency, immune competence, and overall robustness, among others. The term ‘E’ denotes the environmental component of phenotypic variance, which encompasses all controllable external and internal factors, including nutrition, housing conditions, health management, as well as physiological states like age, sex, and reproductive status. The environment can be subdivided into the internal environment (physiological and metabolic status) and the external environment (housing, herd management, climatic conditions). The term ‘e’ accounts for random, uncontrollable environmental effects such as transient temperature fluctuations, accidental pathogen exposures, or other stochastic events that influence the phenotype unpredictably. In the remainder of this review, the term ‘e’ is referred to as the residual error.

### Genes and environment and interactions

**Fig. 2:**
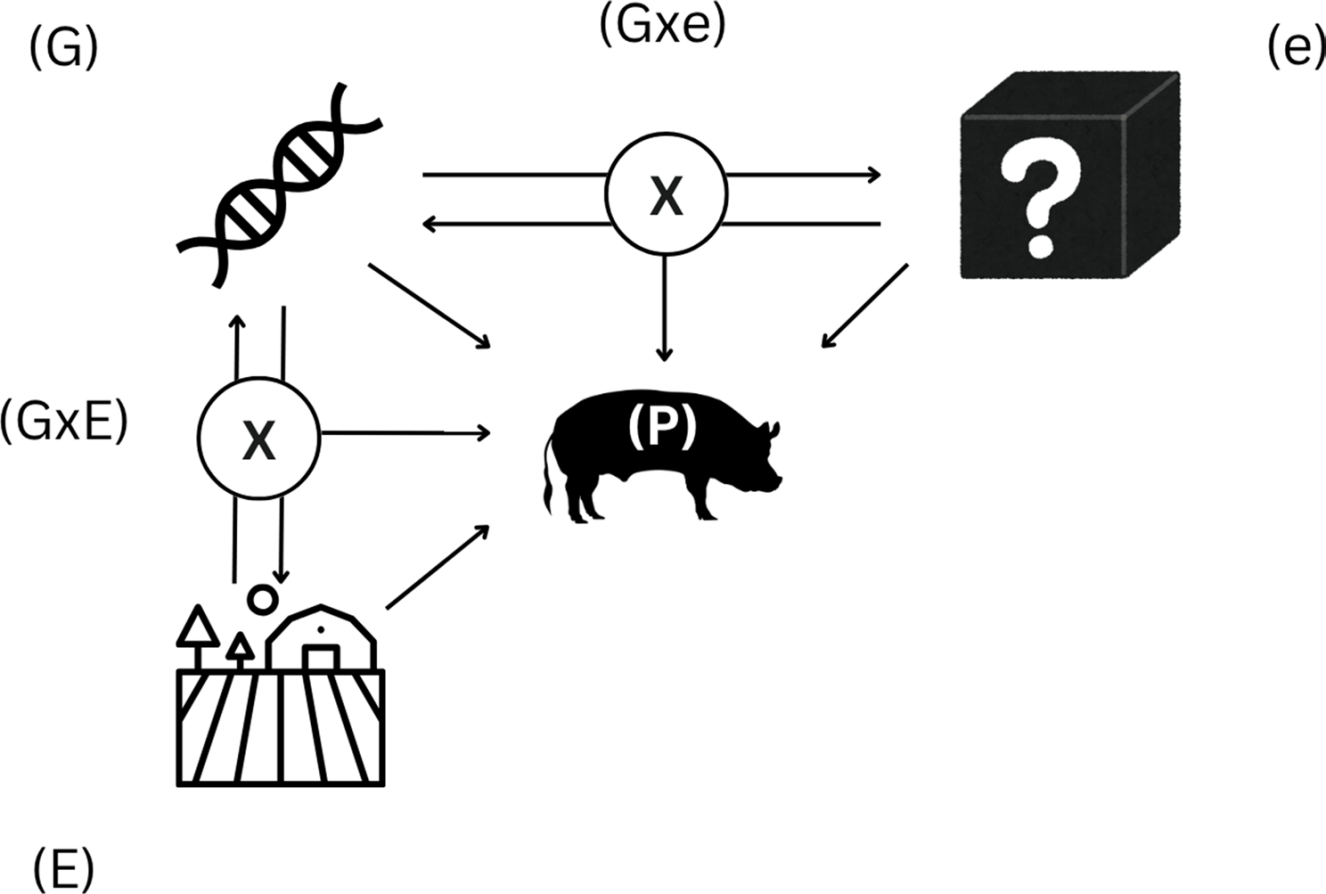
Conceptual model of phenotype (**P**) as the sum of genetic (**G**), environmental (**E**), and random, uncontrollable environmental effects (**e**), plus the interaction of G with E, and interaction of G with e

While this additive model provides a foundational understanding, it overlooks the critical influence of interactions between genetics and environment (Fig. 2). To address this, interaction terms are incorporated:

$${\rm{P = G + E + e + G \times E + G \times e}}$$

The ‘G × E’ interaction reflects genotype-specific responses to controlled and measurable environmental factors; for instance, some pigs may exhibit superior tolerance to heat stress or dietary changes due to their genetic background. As noted above, the residual error (e) represents unexplained phenotypic variation arising from multiple sources i.e., Chen, Shi-Yi, et al. [26]. Conceptually, it can be viewed as comprising two components: truly random variation, such as stochastic biological processes and measurement error, and structured but unobserved effects arising from latent or unmeasured factors. Therefore, ‘G × E’ captures how genetic differences modulate the pig’s reaction ‘*e’.* This relation can conceptualize the host’s genome response to a sum of latent or unmeasured environmental influences. The Gxe and GxE are key to explaining phenotypic variability observed among genetically similar individuals raised under identical conditions or why performance differs across farms with distinct environmental pressures [27].

### Genes, environment microbiota and interactions

**Fig. 3:**
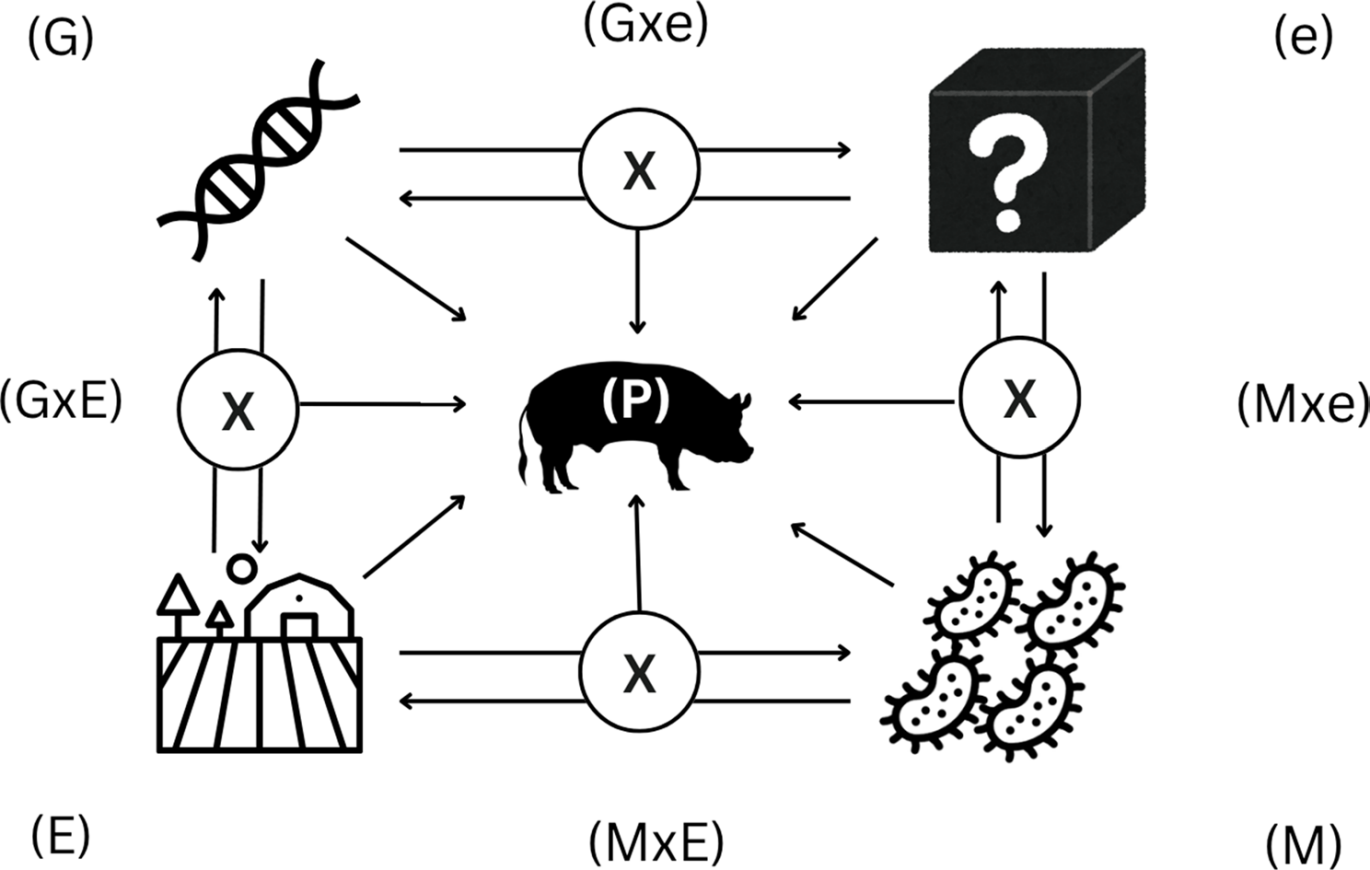
Conceptual model of phenotype (**P**) as the sum of genetic (**G**), environmental (**E**), and random, uncontrollable environmental effects (**e**) and microbiota composition (**M**), plus the interaction of G with E, and interaction of G with e, and interactions of M with E and e

Recent advances highlight the microbiome, the complex community of microorganisms residing predominantly in the gastrointestinal tract, as a significant determinant of phenotype, particularly in relation to resilience and health (Fig. 3).

Thus, the phenotypic equation expands to:

$$\begin{gathered} P = G + M + E + e + GxM + G \times E \hfill \\ \,\,\,\,\,\,\,+ G \times e + M \times E + M \times e \hfill \\ \end{gathered}$$

Here, ‘M’ represents the microbiome’s contribution to the phenotypic variation. The gut microbiota influences host digestion, immune modulation, nutrient absorption, and even behavior, profoundly affecting health and performance. For example, pathogenic bacteria such as *Escherichia coli* can precipitate disease outbreaks, while beneficial taxa contribute to gut homeostasis and immune competence [28].

The microbiome can both shape and be shaped by environmental factors. In the context of ‘M × E’, a given microbiota composition may influence how pigs respond to controlled environmental changes, such as diet or housing modulating their ability to tolerate heat stress or resist pathogens. Therefore, ‘M × E’ can be conceptualized as reflecting how microbial communities may buffer or amplify the host’s response to latent or unmeasured environmental influences, thereby contributing indirectly to residual phenotypic variation without being explicitly identifiable in the model. Importantly, the inclusion of ‘G × M’ interactions acknowledges that host genotypes may differ in their capacity to establish, maintain, or respond to specific microbial communities. Such interactions capture genotype-dependent differences in host–microbiome relationships, whereby the phenotypic effect of a given microbiome configuration may vary across genetic backgrounds.

Together, these interactions highlight that the microbiome is not only a passive recipient of environmental influences (M ← E, M ← e), but also an active mediator of resilience.

Incorporating the microbiome into the phenotypic models enhances our capacity to predict and understand complex traits like resilience, which are otherwise difficult to quantify due to their multifactorial nature and the subtlety of genetic and environmental interactions. It is important to note that genomic-by-microbiome interactions could influence phenotypic variation. However, such interactions were not explored in this study, as the field is still under active investigation and available data in pigs remain limited.

## Measuring resilience in swine: concepts, pros, and pitfalls

The concept of resilience, first introduced by Holling [29] refers to the capacity of a system to absorb disturbances, adapt to change, and maintain essential functions. Unlike stability, which emphasizes a return to the original equilibrium after perturbation, resilience highlights the ability to adjust and continue functioning under stress. Building on this conceptual framework, resilience has become increasingly relevant in livestock production, and particularly in the swine industry, where it reflects the ability of pigs to maintain welfare, health, and performance under variable and challenging conditions [30]. As mentioned before, this growing attention is driven by the intensification of production systems, the need to sustain productivity in suboptimal commercial environments, and mounting societal and environmental pressures, including animal welfare concerns and climate change impacts [3, 31]. Despite its conceptual appeal, resilience remains difficult to implement in breeding programs, as it is not directly observable and must be inferred through proxy indicators. To date, no universally accepted definition or standardized measure of resilience exists, although variance-based metrics, such as the logarithm of phenotypic variance, are increasingly applied in the literature [31]. Consequently, the development and evaluation of robust, biologically meaningful resilience indicators remain critical challenges, particularly under commercial farming conditions. Therefore, for application in animal breeding, an effective resilience indicator should meet several key criteria:

- it should capture biologically meaningful variation related to resilience (relevance)
- possess a genetic component that enables selection (heritability),
- be interpretable and straightforward to apply (interpretability)
- be cost-effective and compatible with large-scale data collection in breeding programs (feasibility).

**Fig. 4:**
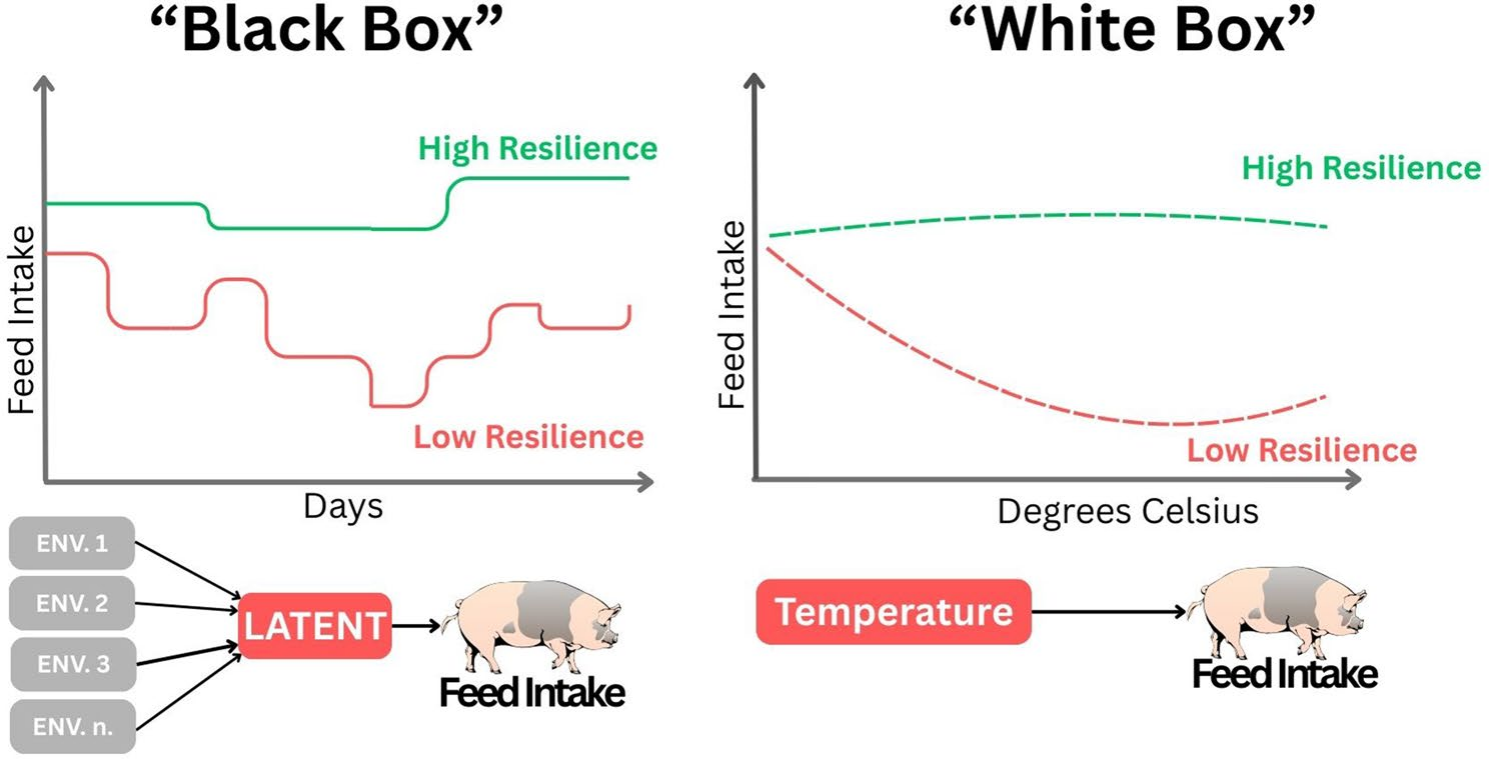
Examples of comparison between black-box and white-box proxy resilience indicators. In the black-box approach, resilience is assessed as fluctuations in a target phenotype (e.g., feed intake), under the assumption that changes are primarily driven by unknown environmental factors. In contrast, the white-box approach evaluates performance changes as direct responses to identified environmental factors (e.g., feed intake variation in relation to temperature). HR indicates the high-resilience group, and LR indicates the low-resilience group

Given these requirements, resilience in pigs is most commonly inferred from production data, which are routinely collected in commercial systems and therefore provide a practical and scalable basis for large-scale application. As illustrated in Fig. 4, production-based resilience proxies can be broadly grouped into two conceptual categories. “White-box” approaches explicitly incorporate information on environmental stressors or challenges, such as genotype-by-environment interactions (G×E), thereby enabling direct assessment of how animals respond to defined perturbations, as discussed in the following section. In contrast, “black-box” approaches estimate resilience from variability in longitudinal performance data without explicitly measuring environmental challenges, treating the environment as a latent factor. Together, these complementary approaches provide practical avenues for integrating resilience into breeding and management programs.

While production-based proxies offer a pragmatic and scalable approach for assessing resilience, particularly within routine breeding programs, they remain indirect measures that capture only the phenotypic expression of resilience. To strengthen their biological relevance, these proxies must be validated against more direct biological indicators related to stress response and health status of the animals. For example, studies by Mancin et al. [32], and Argente et al. [33], have demonstrated associations between variance-based resilience indicators and longevity, disease incidence, and immunological parameters, supporting their relevance to underlying biological robustness.

Direct biological indicators are essential to determine whether observed variation in performance reflects genuine physiological and immunological resilience under both controlled (E) and uncontrolled (e) environmental conditions. To understand the complexity of these direct biological indicators, we need to split the framework into three functional layers:

- disease challenge tests, which provide experimental context to assess animals’ response to stress conditions or controlled pathogen exposure,
- biological indicators, which encompass health status phenotypes, and biomarkers derived from biochemical or immunological assays. These serve as the direct measure of the animal’s internal physiological response; and
- omics-based approaches, including genomics, transcriptomics, and metabolomics, which provide high-resolution insight into resilience mechanisms [34].

Despite their scientific value, these biological approaches are generally too complex, costly, or invasive for routine implementation in breeding programs. Instead, their primary role lies in validating and refining production-based proxies, thereby increasing confidence that simpler, routinely collected indicators meaningfully capture resilience. By offering detailed snapshots of the animal’s biological state, these methods support the use of scalable, production-derived indicators for selection rather than replacing them.

In summary, this section distinguishes between two complementary components:

- Production-based proxies of resilience, including:
- black-box indicators
- white-box indicators
- A biological validation framework, composed of disease challenge tests, biological indicators, and omics, which are used to characterize the biological basis of resilience, serving to validate and support these proxies rather than serving as routine selection criteria.

### Proxy of resilience

#### Black box

Monitoring resilience through production phenotypes is currently the most appealing and extensively discussed approach in livestock systems [35]. This approach is popular for two main reasons: (i) it is cost-effective and (ii) it captures Table 1 resilience in a broad sense. The underlying concept is based on two independent ideas that converge within the same framework, as is explained below.

**Table 1:** Methods used for analyzing genotype by environmental interactions- an example in pigs

Approach	Description
Two trait models	Phenotype splitted into two correlated traits, one for each environment
Reaction norm models	Continuous variation of an environmental gradient. Includes the following approaches
Environmental covariates	Environmental gradient given by contemporary groups of herd, year, season and related effects
Climate covariates	Environmental gradient given by a climatic condition
Multiple environmental covariates	Many environmental covariates included in the model

First, higher variability in observed phenotypes generally indicates lower resilience, as it reflects an animal’s inability to maintain stable conditions under perturbation or external stress. This idea stems from resilience monitoring concepts that originated in the early 1970s aimed at better understanding the dynamics of ecological systems. Although initially rooted in ecology, these concepts have been subsequently adapted for human and livestock systems [36]. The advent of automated data collection technologies, such as automatic feeding systems (AFS) in pigs, has significantly advanced the practical application of these principles in livestock breeding [30]. Foundational insights from early studies have guided the development and critical assessment of modern resilience indicators, particularly when longitudinal data are available [32].

The second idea is based on the concept of environmental variance (VE). Originally defined by Holling [29], VE quantifies the variance in phenotypic values between individuals resulting from differences in environmental exposure [15, 37]. Although VE was not initially conceived as a resilience metric, it aligns with resilience concepts first described by Holling [29] and later expanded by Scheffer et al. [36], as animals exhibiting higher VE are generally more sensitive to (unknown) environmental fluctuations, indicating a reduced ability to maintain stability, an essential aspect of resilience.

##### Early indicators of resilience

**Fig. 5:**
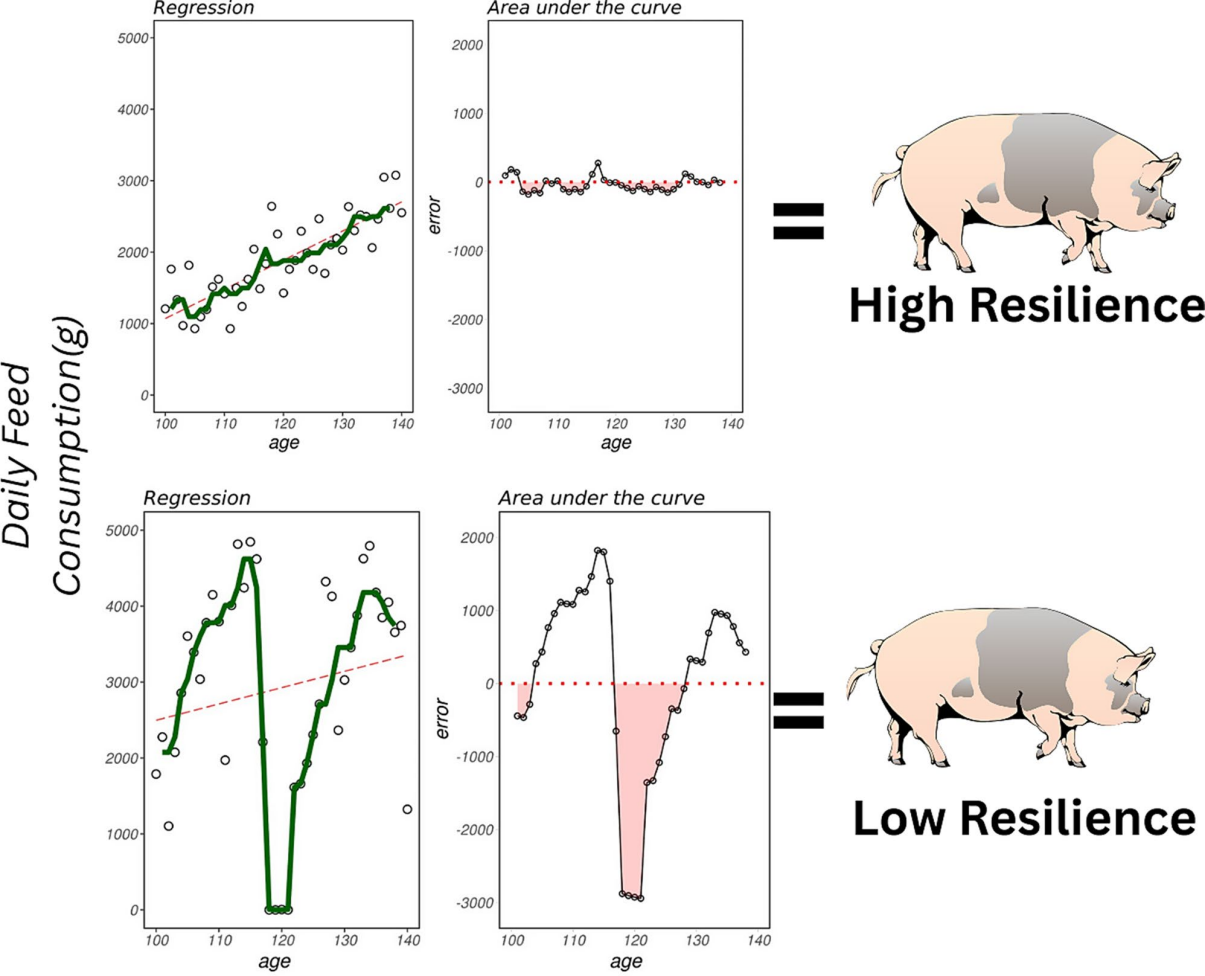
Example of the process for obtaining different “black-box” resilience indicators using feed intake as the target phenotype. The upper part represents animals with low variability (categorized as resilient), while the lower part shows animals with high variability (categorized as non-resilient). The left plot displays predicted values (red) and observed values (green) from spline regression; the variance of differences between the green and red lines was used as a proxy for resilience. The right plot highlights another resilience proxy, showing periods of consecutive negative errors in red. Figure was adapted from Mancin et al. [25],

Holling [29] defined resilience as a system’s ability to absorb disturbances, adapt to changes, and maintain its core functions, distinguishing it from stability, which aims to return to a previous state (i.e., the original state before perturbation). That was represented in Fig. 5. His work introduced key principles that are applicable to livestock systems, including:

- *“Internal” System Variability Enhances Resilience:* Systems with greater internal variability and dynamic fluctuations, such as microbial communities with high alpha-diversity, are often better equipped to adapt to external disturbances. In contrast, overly stable or homogeneous systems may lack the flexibility needed to respond to perturbations, making them more prone to collapse. Importantly, this concept of system variability should not be conflated with variability in system outputs over time (e.g., fluctuations in production performance), as the latter often signals reduced resilience and poor stability.
- *Overmanagement Reduces Resilience:* Increased focus on maintaining stability or maximizing yield can push systems toward critical thresholds, increasing the risk of sudden failures.

Later, numerous studies expanded on Holling’s ideas, exploring the concept of resilience across various contexts. Researchers have focused on decomposing stability into four components: resistance, resilience, persistence, and variability; and have proposed mathematical methods to quantify these aspects. These studies emphasize that resilience, unlike resistance, involves adaptive changes (negative feedback) that enable systems to cope with external stressors [36]. Conversely, resistance is defined just as the capacity to exercise control over the disturbance therefore reducing variability in the output performance.

The concept of resilience has been applied to animal breeding, particularly swine, where researchers broadened the concept to individual organisms, describing them as complex adaptive networks [35]. This approach emphasizes managing resilience at both the organism level and within specific subsystems, such as organs or physiological processes. They introduced *dynamic resilience indicators*, quantitative metrics derived from longitudinal data that detect early signs of declining resilience before a critical transition. These indicators include:

- *Increased Autocorrelation:* Slower recovery from disturbances.
- *Heightened Variance:* Greater fluctuations in physiological or behavioral responses.
- *Enhanced Cross-Correlation:* Stronger interdependence between subsystems’ fluctuations.

This perspective highlights the importance of managing resilience both at the whole organism level and within specific subsystems, fostering a dynamic and adaptive approach to resilience in livestock. In livestock, the relevance of this concept has increased, particularly due to the availability of automated data collection systems that generate large and detailed datasets. These systems facilitate the development of variance-based resilience indicators from routinely collected phenotypes, such as feed intake, growth, or body composition [13]. Recent studies have leveraged high-frequency data from automatic feeding systems (AFS) to develop and validate resilience indicators initially proposed by Poppe et. al., 2020 for automatic miking system [38].

Indeed, the number of studies estimating resilience from AFS data in swine has significantly increased, reflecting a growing interest in this approach. These studies have expanded the proposed metrics range from simpler transformations, as the logarithm of residual variance [39–45], to more complex calculations, including the residual lag [24], maximum days of negative residuals [43], integral of the maximum negative error deviation [40, 44], and differences between expected and observed values

However, the baseline trait used to derive variance-based resilience indicators should be biologically linked to animal fitness. Otherwise, two potential limitations may arise. First, because phenotypic variance often scales with the mean, selection on variance-based indicators may partly reflect differences in average performance rather than resilience per se [42]. Second, for traits not directly related to physiological stability, variability may be difficult to interpret biologically. For example, variation in general activity level could reflect either adaptive flexibility [45] or compromised health, depending on context. These considerations highlight the need for careful trait selection and trait-specific validation when applying variance-based resilience indicators.

Interestingly, some studies have demonstrated that the proposed metrics are indeed connected to resilience, providing robust evidence for their application in animal breeding as phenotypes for resilience [41–47].

##### Environmental variance

VE has emerged as a valuable tool in animal breeding and genetics. As mentioned before, VE refers to the variance in phenotypic values among individuals that arises from differences in their environmental exposures. Interestingly, VE is not solely shaped by external environmental factors, it is also partially influenced by genetic or individual specific factors, as demonstrated by differences in VE among animals raised under the same conditions [15, 26]. In general, animals with lower VE are considered to have a better capacity to maintain stable performance under environmental challenges, reflecting a form of robustness. However, this relationship is context-dependent. In high-management systems, where most environments are relatively uniform, low VE may indicate greater resilience. In contrast, in low-management or variable environments, some degree of phenotypic variability may actually be advantageous, allowing animals to better adapt to changing conditions. This context-dependent interpretation makes VE a promising, yet nuanced, indicator of resilience in animal breeding and genetics.

Furthermore, VE is conceptually related to canalization, which describes an organism’s ability to produce a consistent phenotype despite genetic and environmental variability [48]. This connection underscores the potential of VE to serve as a quantitative measure to evaluate and improve resilience in animal populations. Early methods for estimating VE relied on hierarchical regression models, where residual variance was modeled and assumed to be under both genetic and environmental control [49, 50]. These methods were further refined by [51] using Double Hierarchical Generalized Linear Models (DHGLMs), fitting residual variance using gamma distributions for more robust estimates. VE has been successfully calculated in pigs for various phenotypes, including birth weight [50], body weight at 175 days [49] and litter size [52, 53]. These studies demonstrated that VE is heritable, with heritability ranging from 1 to 10%, suggesting that the opposite of VE could be selected to improve resilience. Notably, two divergent selection experiments validated the genetic control of VE: Formoso-Rafferty et al. [54] on VE of birth weight in mice using Bayesian DHGLM, and Blasco et al. [55] on VE of litter size in rabbits using a simpler, more pragmatic procedure that avoided highly parameterized models and potential mathematical artifacts. This latter approach computes the variance of residual errors for each animal and treats it as a new phenotype. In both cases, the selected resilience indicators were associated with physiological markers of resilience as well as survival, longevity, and overall fitness, supporting their biological relevance.

Interestingly, this pragmatic approach aligns with the concept proposed by Holling [29]. Although Sell-Kubiak et al. [53] demonstrated that the approximate two-step approach and hierarchical methods are not fully equivalent when applied to litter size in pigs—reporting a correlation of 0.87 between breeding values—the hierarchical method generally provides more accurate estimates. Nevertheless, the two-step approach has gained popularity in high-throughput data contexts, such as those involving AFS [26], primarily due to its interpretability and methodological flexibility.

##### Pros and cons of estimating resilience from production phenotypes

Building on Holling’s [29] foundational definition of resilience and Falconer’s concept of VE, monitoring variability is a bit clearer in target phenotypes has become a widely adopted proxy for assessing resilience in swine production, particularly when using large amounts of longitudinal data. One of the main advantages of this approach is its cost-effectiveness, as it leverages routinely collected phenotypic data to estimate resilience. However, despite these benefits, several challenges remain.

A key limitation is that resilience-related deviations can be masked within large datasets, especially when the target phenotypes, such as certain production traits in swine, have only a weak correlation with overall animal fitness [43]. In some cases, observed fluctuations might not directly indicate reduced resilience; instead, they could represent adaptive responses aimed at maintaining internal stability when faced with external perturbations. For example, in dairy animals, variability in milk production can act as an adaptive mechanism to conserve energy for vital functions (e.g. reproductive function), rather than indicating reduced fitness. According to Holling’s framework, resilience is the capacity to adapt and persist under changing conditions, whereas stability refers to returning to an initial state after a disturbance. Thus, excessively minimizing variance may reduce a system’s adaptability, as some variability is essential for resilience.

However, this concept does not universally apply to all species. In pigs, traits like feed intake are more directly associated with fitness, and fluctuations in these traits might either be neutral or genuinely linked to resilience. Therefore, interpreting variability in pig traits requires careful consideration of the specific biological context and the trait’s relationship to overall fitness and resilience [43]

Another challenge, albeit less critical than the first, is that large datasets can also introduce complexities related to random fluctuations that are not biologically meaningful, such as those unrelated to fitness or health. These random variations can obscure true resilience measures if not properly accounted for, potentially capturing only the mean phenotype rather than genuine resilience. Moreover, since variance is inherently correlated with the mean, selecting for reduced variance could inadvertently result in selecting against the mean of productive traits. Tatliyer et al. [56] demonstrated this issue through simulations, revealing that positive genetic correlations between the mean and variability of traits were partially influenced by scale effects, where higher means were associated with greater variances. These correlations ranged from 0 to 1, highlighting the potential risks of mean-based selection.

Furthermore, Mancin et al. [42], conducted genome-wide association studies analyzing variance in daily feed intake while accounting for random fluctuations. When random fluctuations were considered, the genes identified were similar to those associated with average feed intake. In contrast, excluding random fluctuations revealed different genes related to immune function and PRRS infection. This distinction underscores the importance of appropriately handling random fluctuations in resilience studies to avoid misleading genetic interpretations. For a more comprehensive assessment of resilience, and to improve accuracy, alternative indicators that go beyond residual variance should be considered, such as those proposed by Gorssen et al. [46] and Laghouaouta et al. [47], considering the natural logarithm of the variances of observed versus predicted body weights, or the deviation of a linear interpolation of body weight from expected production levels.

#### White box

Genotype × environment (G×E) interactions are commonly used to describe macro-environmental sensitivity, defined as changes in the mean phenotypic performance of genotypes across contrasting environments. In this context, phenotypic expression is assumed to depend on the type of environment to which individuals are exposed, and genetic differences in environmental response are often interpreted as an expression of resilience under controlled or defined conditions. As clearly outlined by Jinks et al. [57], and further formalized in Ros et al. [58], environmental sensitivity can be defined in two distinct but complementary ways: either as mean phenotypic changes of a genotype across environments, or as differences in residual phenotypic variance among genotypes within the same environment. These two definitions give rise to different statistical modeling frameworks.

Under the first definition, environmental sensitivity is modeled through genotype-specific responses to environmental change, typically considering discrete or continuous environmental gradients such as low versus high temperature. In this framework, phenotypic plasticity is described by including a genetic component in a regression of phenotype on the environment, commonly implemented using reaction norm models. Differences among genotypes are therefore expressed through variation in both average performance and sensitivity to environmental conditions.

In contrast, the second definition of environmental sensitivity focuses on micro-environmental sensitivity, which is reflected in genotype-specific differences in residual variance (Vₑ) among individuals exposed to ostensibly identical environments. Here, a given genotype is expected to have a characteristic mean phenotype, but observed values deviate from this expectation due to the cumulative influence of numerous, often unmeasured, environmental factors experienced throughout the individual’s life. These deviations are modeled as random residual noise, whose variance may depend on both covariates and genetic effects, allowing different genotypes to differ not only in mean performance but also in environmental (residual) variability.

From a biological perspective, pigs are continuously exposed to a wide range of environmental influences, including climatic stressors such as heat and humidity as well as differences in farm management practices, all of which can affect performance. Under challenging conditions, such as elevated ambient temperatures, individuals often exhibit declines in productivity and welfare [59]. The ability of pigs to maintain stable production across such environmental fluctuations is therefore commonly regarded as an expression of resilience.

Traditionally, G×E has been investigated by splitting environments into discrete categories and analyzing the genetic variability of traits under contrasting conditions, for example, temperate versus tropical climates or conventional versus organic farming systems [60, 61]. This approach provides insights into how pigs cope with different levels of environmental stress, highlighting those genotypes that perform consistently across environments. However, it assumes only a few distinct environmental types, which may not fully capture the continuous nature of environmental variability.

More recently, reaction norm models have been increasingly used to study resilience. These models describe how an individual’s performance changes along an environmental gradient, treating the environment as a continuum rather than as discrete categories [62]. In this context, the slope of the reaction norm represents the animal’s sensitivity to environmental change, while the intercept reflects its average performance. Animals with flatter slopes can be considered more resilient, as their performance is less affected by environmental fluctuations, whereas steeper slopes may indicate higher sensitivity.

Environmental descriptors for such analyses include farm-level factors (e.g., herd-year-season effects) and climate variables such as temperature and humidity, often summarized in indices like the temperature-humidity index (THI) [63]. In pigs, heat stress is a particular concern due to their limited thermoregulatory capacity [64], and studies have shown that resilience to heat stress can vary among genotypes [65]. The availability of climate data and farm records enables these models to assess resilience using routinely collected information, making them practical for breeding programs [66].

By incorporating G×E effects, breeding values can be adjusted to account for macroenvironmental sensitivity. This adjustment helps identify pigs that are not only high-performing but also capable of maintaining their performance under varying conditions. Selecting for such traits is particularly relevant in the face of climate change and the increasing variability of production environments. While resilient animals maintain stable production, others may exhibit plasticity, altering their performance in response to environmental changes. The choice between resilience and plasticity as breeding goals depends on the production system: stable environments may benefit from plastic animals able to maximize their genetic potential when conditions are optimal, while variable or challenging systems require resilient individuals less affected by environmental stress [67]. A resume of all methodology of GxE was reported on Table 1.

Understanding G×E interactions in pigs thus provides a valuable tool for monitoring resilience (specifically macro-environmental sensitivity M x E) and guiding breeding decisions toward animals better suited to future farming challenges

### Validation approaches for resilience

Previous studies are valuable for breeding applications because they allow resilience to be quantified and selected. However, these approaches rely on phenotypic assumptions i.e. such as equating higher variability with lower resilience, rather than direct biological definitions. Consequently, biological validation remains essential.

#### Challenge tests

Disease challenge tests involve the intentional exposure of animals to specific pathogens under controlled conditions to evaluate their physiological, immunological, and performance responses. In swine, such tests have been extensively employed to assess resilience, resistance, and tolerance traits, particularly in the context of vaccine development and the optimization of health management strategies aimed at minimizing productivity losses [68]. By replicating defined pathogenic stress scenarios, challenge trials provide a robust framework for disentangling the genetic and phenotypic determinants of variation in host responses. This approach aligns closely with the operational definition of disease resilience: the ability of an animal to survive, recover, or maintain functional output when exposed to infectious pressure.

**Fig. 6:**
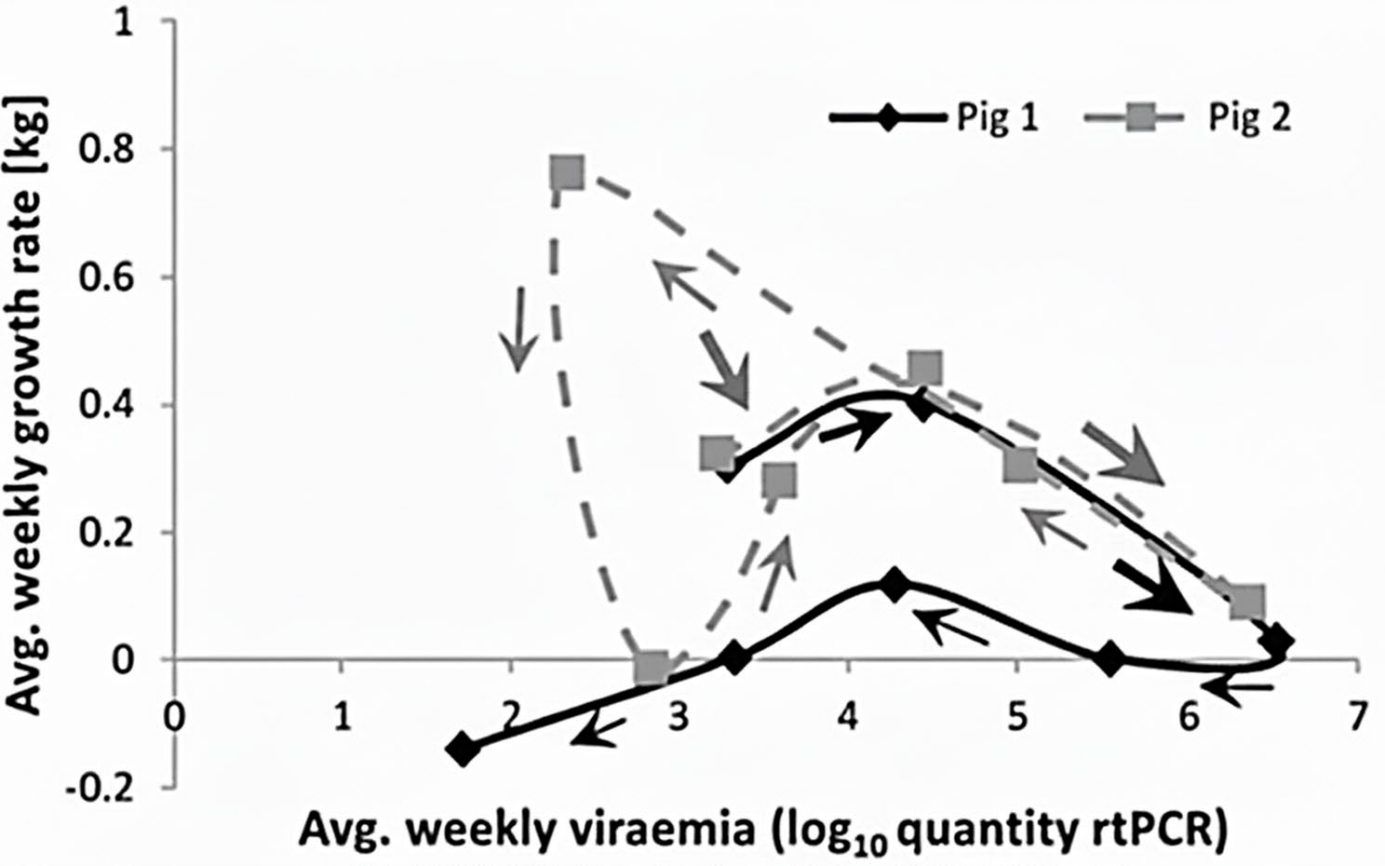
Performance (average weekly growth rate) vs. Pathogen burden (average weekly viraemia) trajectories for two individual pigs infected with the same challenge dose of a virulent PRRS virus strain. Arrows indicate the direction of growth rate-viraemia plots over time. The size of the arrows crudely reflects the speed at which the trajectory progresses.Figure from Doeschl-Wilson et al. [73]

Disease resilience is inherently complex, as it encompasses dynamic and transient host immune responses that modulate interactions between pathogens and their hosts [69]. Conceptually, resilience integrates two core mechanisms: resistance, defined as the ability to limit within-host pathogen replication, and tolerance, the capacity to sustain performance despite high pathogen burdens [14]. Notably, disease challenge tests have also provided critical insights into pathogen-specific host responses. For example, intravenous inoculation with *Mycoplasma hyorhinis* reliably induces visible lesions, creating a controlled model for studying disease pathology and host responses [70, 71]. Similarly, intratracheal challenges with *Haemophilus parasuis* have been used to distinguish between acute and chronic disease states, depending on the timing and type of samples collected [72]. Beyond pathogen-specific outcomes, these controlled models provide an unique opportunity to validate the production-based proxies previously discussed. For example, Putz et al. [43] demonstrated that deviations in feed intake under infectious pressure can serve as a practical proxy for disease resilience, reflecting an animal’s ability to maintain intake despite systemic stress. To capture disease resilience complexity, researchers increasingly employ longitudinal measurements of health and performance to construct Health-Performance Trajectories (HPTs). These trajectories plot individual animals’ performance metrics (e.g., growth rate) against pathogen burden or immune response indicators over time (Fig. 6), offering dynamic insights into resilience phenotypes [74, 75].

The shape of these trajectories reflects distinct resilience patterns. Resilient individuals that recover and return to high health and performance levels tend to form looping trajectories, with smaller loops indicating faster recovery and less perturbation [76]. While this framework has gained considerable traction in human medicine [73, 77], its application in livestock remains largely theoretical [78, 79]. However, such findings highlight the potential of challenge trials not only for identifying phenotypic indicators but also for validating predictive resilience models derived from longitudinal production data, thus bridging experimental and field-based approaches.

Despite their scientific value, disease challenge tests have notable limitations. High-resolution phenotyping often requires invasive, labor-intensive, and costly procedures, limiting scalability. Additionally, most challenge models are pathogen-specific, which reduces their relevance to the diverse and evolving disease landscapes typical of commercial farming systems [80]. Although immunological assays provide detailed mechanistic insights, their high cost and logistical demands hinder their routine use in large-scale breeding programs.

These constraints have fueled growing interest in less invasive, more scalable alternatives for assessing resilience. By identifying high-throughput phenotypic proxies, researchers aim to enhance robustness and disease resistance in pig populations, reducing reliance on resource-intensive challenge trials while supporting sustainable production goals [81].

However, disease challenge tests are the most commonly applied form of controlled stress exposure, although they represent only one of several possible classes of challenge tests. More generally, challenge tests may involve other types of stressors, such as social or behavioral challenges linked to management practices, including stress induced by social mixing, as well as thermal challenges, which are increasingly relevant in the context of climate change aforementioned. In pig production, nutritional challenges are of particular relevance, as the ability to cope with diets higher in fiber or fat,including those derived from human food waste, is becoming increasingly important. This form of “feed resilience” is likely to gain importance in the context of rising food–feed competition and the development of circular feeding systems, making nutritional challenge tests a valuable complement for validating resilience indicators.

#### Biological indicators

Biomarkers serve as indirect indicators of resilience by predicting how swine respond to various stressors. Because they are typically measured as continuous variables, biomarkers enable quantification of the stress response, facilitating more nuanced assessments compared to binary health outcomes.

Among available biomarkers, blood cell traits have emerged as valuable proxies for resilience and are increasingly considered as potential selection criteria in breeding programs. Blood cell biomarkers can be broadly classified into three main categories:

i. Mitogen Stimulation Assays: These measure the proliferation of peripheral blood mononuclear cells following stimulation with mitogens such as concanavalin A, hemagglutinin, or phorbol myristate acetate. These assays have shown moderate correlations with disease resilience traits [82], suggesting their potential as genetic indicators for resilience and as selection traits within breeding schemes [79].
ii. Complete Blood Count (CBC) traits: This category includes lymphocyte counts and hemoglobin levels, which are heritable and genetically correlated with key performance metrics such as growth rate and treatment response, underscoring their value in resilience-focused selection programs [13, 83]. Specifically, Bai et al. [78] demonstrated the practical utility of this category by showing that hematological parameters can differentiate pigs with faster and more efficient immune responses, identifying individuals with higher resilience potential.
iii. Phagocytosis Assays: These evaluate the functional activity of immune cells,particularly eosinophils. Enhanced phagocytic activity has been linked to reduced mortality and improved growth performance, supporting its use as a potential indicator for selecting resilient animals [84].

However, interpreting immune-related biomarkers requires careful differentiation between resistance and tolerance mechanisms. A robust immune response typically indicates resistance, the host’s ability to limit pathogen replication, whereas a moderated or lower immune response may reflect tolerance, where the animal maintains performance despite infection without incurring the costs of excessive immune activation. Thus, a low immune response can, in some cases, signify resilience through tolerance rather than a lack of resistance. Recognizing this distinction is critical to the effective use of immune biomarkers in resilience assessment and breeding decisions.

In this context, health status phenotypes such as mortality, morbidity and clinical lesions provide a comprehensive assessment of animal resilience. Establishing correlations between these broad health indicators and the previous performance-based proxies is key to ensuring that observed resilience reflects health rather than statistical artifacts. For example, Gorssen et al. (2024) [13] reported low to moderate positive genetic correlations between deviations in longitudinal weight data and health phenotypes such as mortality, tail biting woundHs and lameness. Similarly, Casto-Rebollo et al. (2025) [85] demonstrated the practical implications of this relationship, showing that progeny from non-resilient boars exhibited a 2.5% higher mortality rate compared to those from resilience counterparts. These findings underscore that direct biological indicators are essential to confirm that an animal’s capacity to maintain performance is grounded in superior health and efficient immune response. These outcomes serve as the biological anchor required to validate performance-based proxies of resilience within commercial livestock systems.

#### Omics-based approaches

The recent advent of omics technologies, such as transcriptomics, proteomics, and metabolomics, has broadened the search for resilience biomarkers by providing molecular-level insights necessary to elucidate the underlying mechanisms that connect an animal’s genotype with its complex phenotypes [86]. Rather than being immediate diagnostic tools, omics approaches facilitate the identification of molecular signatures and biological pathways associated with resilience traits that are otherwise hidden within macroscopic data [87]. Hence, the primary utility of these technologies lies in their ability to deconstruct the “black box” of resilience, revealing the specific metabolic and signaling pathways that allow an animal to cope with stress.

Once these mechanisms are characterized, specific metabolites, transcripts or genetic markers can be validated and subsequently employed as precise resilience biomarkers. For instance, Mancin et al. [42] and Casto-Rebollo et al. [88] utilized GWAS to link production-based proxies from feed intake records with genes involved in immune-related pathways. By uncovering candidate genes of resilience, these studies are the first steps to identifying genetic markers that could eventually serve as biomarkers for selection. This demonstrates how omics data can capture resilience-related responses beyond immune function, including adaptations to environmental stressors such as heat or combined stresses [29].

Nonetheless, employing omics data as direct indicators of resilience presents significant challenges. Resilience is a highly complex, dynamic phenotype involving numerous biological systems, including the nervous system, sensory perception, and immune responses. Identifying specific genes, metabolites, or proteins responsible for resilience is complicated by extensive interactions among multiple overlapping pathways. Furthermore, resilience is context-dependent and can vary over time, making it difficult to isolate consistent molecular markers.

Consequently, while omics technologies offer promising avenues to deepen our biological understanding of resilience mechanisms, their practical application as reliable resilience indicators currently lagging behind more established proxies like blood cell traits or production data. Advancing this field will require sophisticated analytical methods and further research to elucidate the complex molecular architecture underlying resilience and to develop robust omics-based biomarkers suitable for breeding applications.

Within the omics spectrum, the gut microbiome has attracted considerable interest as a potentially informative resilience biomarker due to its well-established links with animal health, performance, and adaptability [89]. Influenced by both host genetics and environmental factors, the microbiome represents a dual-faceted entity, acting as both a marker of resilience status and a modulator of host physiological responses [25]. However, as with other omics approaches, microbiome-based indicators remain exploratory. Significant research is still needed to define reliable associations between specific microbial features and resilience phenotypes. The inherent complexity of microbiome-host-environment interactions necessitates cautious interpretation and underscores the importance of continued investigation, which will be further addressed in the following section.

## Microbiota and holobiont in swine

### The holobiont: what is it ?

The genetic components of the host and the gut microbiota, as mentioned in the previous section, can be viewed as a single integrated unit, commonly known as the *holobiont*. The roots of the holobiont concept date back to the 19^th^ and early 20^th^ century, when it was hypothesized that mitochondria and chloroplasts could have been symbiotic bacteria that are now systematically transmitted through the cytoplasm of the host’s cells [90, 91] defined the holobiont as “*a multispecies consortium of cells with many genomes that can contribute to multiple functions throughout the body*.” This definition captures the symbiotic relationship between the animal host and its microbial communities, where both the host and microbial genomes interact and contribute to the overall function and phenotype of the organism. The host genome not only regulates its own cells but also influences the composition and activity of the gut microbiota. In contrast, microbial genomes can modulate their own gene expression and influence host physiology and traits. From a host genetic perspective, the holobiont represents a significant extension of the coding potential beyond the host’s chromosomal DNA [92].

A key implication of the concept of holobionts is that many bacterial symbionts are not transient external entities but enduring functional components that reside within the host. These microbes occupy specific niches inside the host’s body, particularly within the gut, and contribute to host biology in a way that parallels organ systems or genetic elements [93].

### The holobiont and microbiota: formation, function, and transmission

The concept of holobiont emphasizes that animals and their associated microbial communities form an integrated biological unit whose formation, stability, and transmission are essential for host development, physiological function, and adaptive capacity. The establishment and persistence of the holobiont depend on a sequence of temporally structured conditions:

1. *Holobiont formation:* there is presence of abundant and diverse microbiota in the environment, allowing the microbiota to enter in symbiosis with the host.
2. *Microbiota host connection:* once the symbiosis is established, this will have an impact on the host fitness. The microbial-host interaction must confer a measurable benefit on the host, such as improved nutrient metabolism, immune readiness, or stress buffering, factors that directly contribute to resilience.
3. *Trans-generational fidelity:* in order to maintain these benefits over time, the symbiont must be consistently transmitted from the parent to the offspring, preserving the functional structure of the holobiont between generations.

Importantly, the establishment of a specific symbiont is not random. Host genetics plays a crucial role in shaping the microbiota [94], as the internal host environment provides selective pressures that favor colonization of preferred and functionally beneficial microbial partners [95]. This genetic-microbial interplay further supports the heritability of resilience traits, extending beyond the host genome to include the microbiome as part of the total heritable phenotype.

This happens by means of:

*Obligate symbiosis:* These microbes can only survive within the specialized environment provided by the host [96]. Due to long-term coevolution, many of these species exhibit genome reduction and loss of certain functions, reflecting adaptation to a highly specific ecological niche [97, 98].

*Host-dependent symbiosis:* In some cases, hosts require specific microbial species for essential biological functions. This type of dependency is especially pronounced in invertebrates [99, 100].

*Partner fidelity:* This term refers to the evolutionary stability and specificity of host–symbiont relationships, where the same symbiotic partners are maintained across generations due to mutual benefit and coadaptation [101].

It is also important to distinguish between the true symbiont and other microbial associates based on their interaction with the host. For example, *Mycobacterium tuberculosis* can persist in the host for decades without causing overt disease and, under broad definitions, can be considered a symbiont. In contrast, *Vibrio cholerae* causes acute, often lethal disease shortly after infection and therefore does not qualify as a symbiont [102].

Together, these classifications support the view that the host and its microbiome form a functional, co-evolving unit. A key aspect of this concept is the intergenerational transmission of microbial communities [103]. This perspective allows the microbiome to be considered a heritable trait of the host species, analogous in consistency and biological relevance to traits inherited through Mendelian genetics.

Four basic principles have been recognized as the basis of the Holobiont coevolution, or the so-called “hologenome concept of evolution” [94].

(i) All animals and plants host a variety of microbiota, and therefore they are holobionts.
(ii) The holobiont works as a distinct biological entity from an anatomical, metabolical and immunological point of view during development and evolution.
(iii) A significant fraction of the hologenome, microbial genome plus the host genome, is transmitted over generations, therefore propagating the unique features of the holobiont.
(iv) Both the changes in the host genome and the changes in the microbiome genome may produce genetic variation in the hologenome.

Being the microbiome faster in its environmental dynamics and processes than the host genome, it can play a key role in the holobiont’ adaptation and evolution. Consistent with this view, in pigs, differences in microbiome composition have been observed among breeds, indicating that host genetic background interacts with the microbiome to shape breed-specific adaptive responses [104].

Hence, the holobiont shows an undeniable connection to the animals’ phenotypes. [105]. In mammals, the microbiome, in interaction with host genetics, is particularly important for physiological processes related to phenotypic plasticity, dietary flexibility, digestive efficiency, and immune modulation [106, 107]. Together, these functions support the animal’s ability to maintain health and productivity under fluctuating or stressful conditions, collectively referred to as resilience. Additionally, microbiome-mediated effects might persist across generations. In swine, vertical microbial transmission occurs when piglets acquire key symbionts from the sow during birth and early-life contact, including microbes involved in immune development and metabolic programming [108]. When such microbial communities are consistently transmitted and maintained, they contribute to the continuity of holobiont function and may reinforce resilience-related traits as part of the animal’s extended, heritable phenotype.

### How holobiont is formed and sustained in swine

In swine, microbial colonization represents the first essential step in the establishment of the holobiont. This foundational phase initiates the symbiotic relationship between the host and the microbiota and has long-lasting implications for host development, resilience, and health. Current evidence in mammals suggests that, in healthy pregnancies, stable gut microbiota colonization largely begins during and immediately after birth [109], while any microbial presence or exposure in utero, if present at all, is likely limited and may primarily reflect microbial components or metabolites that prime fetal immune development rather than an established intestinal microbiome [110].

**Fig. 7:**
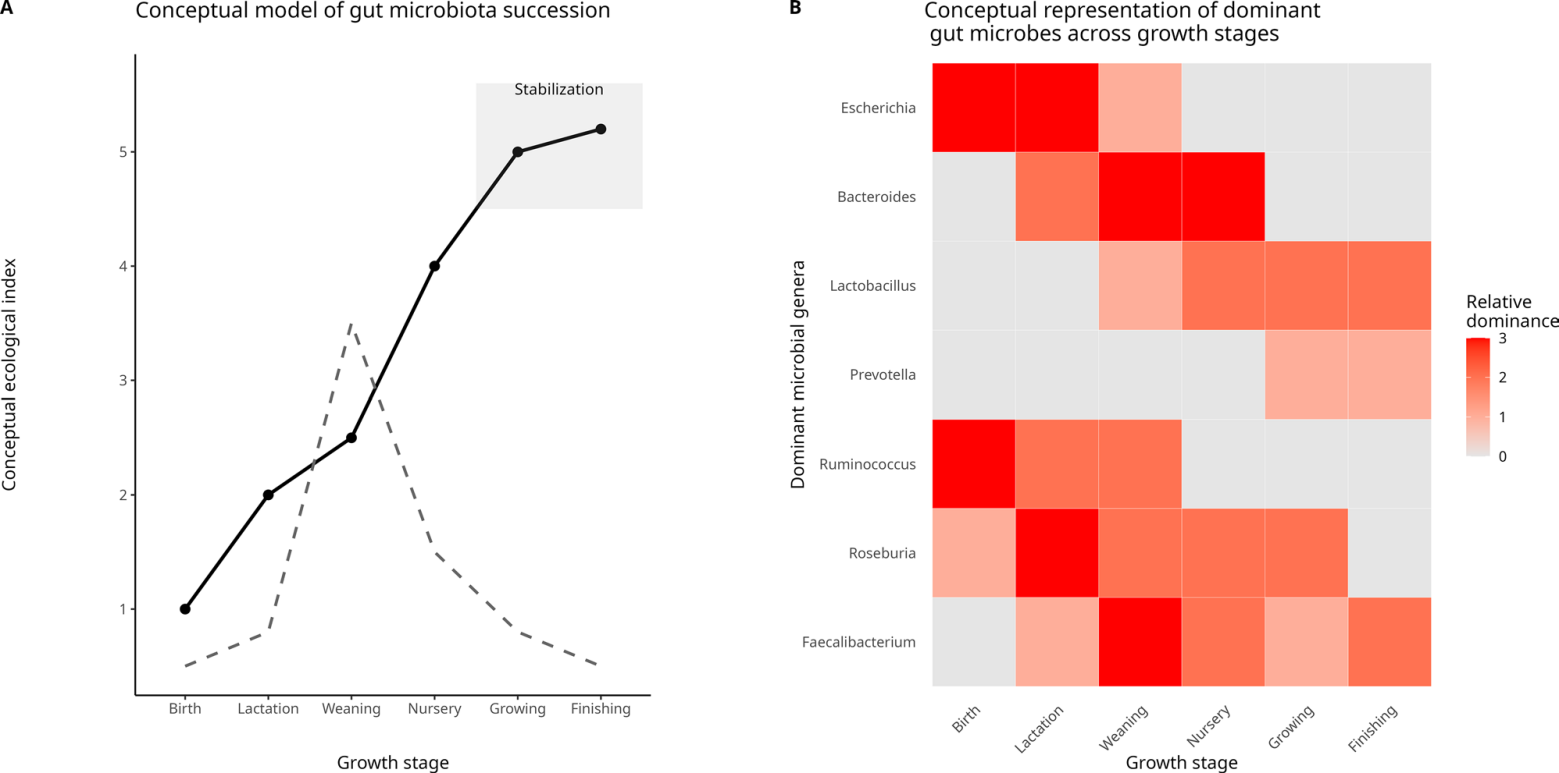
Conceptual representation of gut microbiota succession across pig growth stages. (**A**) Age-dependent changes in gut microbial diversity and community turnover. The solid line represents progressive increases in microbial diversity, while the dashed line indicates species turnover, highlighting pronounced community restructuring during the weaning transition and subsequent stabilization at later growth stages. (**B**) Qualitative representation of stage-specific dominance of major gut microbial genera across developmental periods, illustrating early-life colonization by pioneer taxa and the emergence of a mature, functionally stable microbiota during growth and finishing stages. All trends are shown conceptually to illustrate ecological patterns rather than quantitative abundance. Conceptual figures were generated in R using ggplot2 to illustrate ecological patterns of microbial succession, turnover, and stabilization across growth stages. Values shown do not represent empirical measurements but are intended to support conceptual interpretation

The composition of the symbiont microbiota, and thus the holobiont, is known to change dynamically throughout the life of the host. However, the trajectory of these changes can vary significantly between individuals and species. Gut microbial community assembly in pigs follows a clear age-dependent succession pattern, characterized by progressive increases in community complexity and diversity across the host’s lifespan, Fig. 7. Early life is dominated by pioneer taxa such as Escherichia, Bacteroides, and Clostridium, reflecting initial colonization processes occurring around birth and early environmental exposure [111, 112]. During lactation, milk-oriented microbial communities are enriched in Bacteroides and Lactobacillus, while overall microbial diversity remains relatively low [112]. Typically, a rapid increase in microbial richness is observed during early life, followed by stabilization toward a relatively mature and functionally stable gut microbiota as animals approach adulthood [112]. The transition to weaning represents a major ecological perturbation, marked by pronounced community disruption and dietary-driven restructuring, with a decline of milk-associated taxa and the rapid emergence of Prevotella as a dominant genus [111, 112]. Post-weaning and nursery stages are characterized by increasing microbial diversity and progressive stabilization of the gut ecosystem, accompanied by the expansion of fiber-degrading and short-chain fatty acid–producing genera such as Prevotella, Ruminococcus, and Roseburia [112]. As pigs progress into the growing stage, the gut microbiota becomes more mature and resilient, exhibiting higher diversity and functional redundancy, with sustained dominance of Prevotella alongside genera such as Faecalibacterium and Ruminococcus [111, 112]. These age-dependent dynamics are schematically illustrated in Fig. 7 using a synthetic dataset designed to reflect the ecological patterns described in [111, 112].

In adulthood, the gut microbiota is thought to consist of the following major components:

- *(a) Core microbiota:* A conserved set of microbial species shared between individuals of the same host species (e.g., swine), likely essential for basic host functions.
- *(b) Environmentally acquired microbiota:* A host-specific microbial pool shaped by individual environmental exposures, such as housing conditions (e.g., indoor vs. outdoor systems).
- *(c) Flexible microbiota:* A more plastic component capable of responding rapidly to short-term environmental changes, such as dietary changes (e.g. transition from gestation to lactation diet).

This adult microbiota is generally more resistant to disturbances compared to young animals, mainly due to the stabilizing influence of the core component (a). However, individual differences emerge from environmental influences (b), and within-individual temporal changes may occur due to the flexibility of component (c).

From a taxonomic perspective, while the overall composition may be conserved at higher taxonomic levels (e.g., phylum or family), the variation is more pronounced at the genus and species/strain levels, due to the more specific functionality [113].

Finally, life progression can be interpreted as continuous microbial exposure. Under the theoretical assumption of an infinitely extended lifespan, it can be expected that the host will encounter every possible contemporary environmental microbe, leading to an ever increasing microbial richness over time [114].

The establishment and evolution of the swine gut microbiota occurs through distinct colonization stages, each shaped by developmental milestones and environmental exposures.

#### In-utero colonization

It is still under debate whether a fetus is sterile prior to conception. Although earlier studies suggested that the fetal environment was sterile [115], more recent research indicates that bacteria can colonize the host’s intestines before birth [116]. Bacteria have been detected in the umbilical cord, amniotic fluid, and even the bowels of the newborn, suggesting that bacteria can cross the placental and amniotic barriers [108]. In poultry, for example, pathogens such as Salmonella and Mycoplasma may be transmitted to the offspring, not only from the eggshell but also from the egg yolk [117].

#### Colonization at birth

The transfer of bacteria to amniotic fluid, combined with the inevitable ingestion of amniotic fluid by the piglet, suggests the passage of bacteria to the piglet’s intestines [118]. Furthermore, as the newborn passes through the birth canal, it is exposed to the microbial communities that inhabit the canal, facilitating the colonization of the newborn.

Thus, the initial colonization of the newborn’s intestines is largely the result of vertical transmission from the dam. The sow, through her body and physiology, regulates which microbes can be passed on to the offspring. For example, studies have shown that the vaginal tract of the sow exhibits an increase in the abundance of *Lactobacillu*s spp. around gestation, which likely aids in the establishment of this species in the piglets’ intestines [119].

#### Colonization during later life

During the lifetime of the animal, vertical transmission gradually gives way to horizontal transmission. Weaning represents a significant turning point, as piglets are separated from their mothers, no longer share the same environment, and a solid diet is incorporated. However, in cases where multiple generations coexist in the same environment, some parent-offspring microbial transfer may still occur. For example, in cattle, the calves’ rumen can become colonized by the fecal or ruminal microbes of their parents when calves graze on grass where saliva or feces of their parents are present [107]. A similar mechanism may occur in pigs, whether raised indoors or outdoors, if they share the same environment to any extent [120].

As animals mature, horizontal transmission becomes more frequent and important [106]. This process depends not only on the animal’s exposure to microbes but also on the necessity of acquiring certain microbes. From a symbiotic perspective, Alberdi et al. [103] proposed that not all symbionts are equally likely to be acquired horizontally. Obligate symbionts are typically passed vertically, obligate anaerobes are mostly acquired vertically, while obligate aerobes are more likely to be acquired horizontally. This suggests a limited window of opportunity for the acquisition of certain microbes, influencing the resulting microbial richness in the host. In this context, the strategic use of probiotics and prebiotics becomes important [121]. Furthermore, the established microbiota itself can limit the colonization of new microbes. Existing symbionts can produce antibiotics that protect their niches, thus protecting the host from invasion by pathogens or competing symbionts [122, 123]. Early colonizers, acquired vertically, tend to occupy niches in the gut and prevent other microbes from establishing themselves to some extent. This highlights the importance of vertical transmission of beneficial symbionts from the sow to the piglets, likely leaving a long-lasting impact on the health of the piglets.

Although the microbiome encompasses diverse microbial communities throughout the body, in this context we focus specifically on the gut microbiome, as it has the most direct link to host physiology and the largest body of literature related to resilience in livestock. Accordingly, the next section will focus exclusively on the gut microbiome.

## Holobiont and microbiota links with resilience in swine

As discussed previously, the microbiota, along with the host’s genetic makeup, plays a key role in the shaping of animal phenotypes, including traits associated with resilience [110]. While the influence of host genetics on resilience has been extensively studied, both in human and livestock, the role of the microbiota has only recently gained significant scientific attention. As described in the holobiont section, the microbiota actively contributes to the host phenotype through its symbiotic relationship with the host’s genetics, allowing the modulation of various physiological processes [105]. Particularly in swine, the joint influence of the microbiota and host genome on the phenotypes, is increasingly investigated through the concept of *microbiability*, that is the proportion of phenotypic variance statistically attributed to the microbiota [124]. Although microbiability does not imply direct causation, it reflects the degree of association between microbial composition and host phenotype. Studies have shown that the microbiota can explain between 20 and 35% of the variation in performance traits such as feed intake and average daily gain [92, 125]. However, other studies have reported much lower estimates of microbiability, in some cases close to zero [21]. These findings underscore the significant impact of microbial communities on swine productivity, as shifts in their composition are often linked to changes in performance outcomes. Despite growing recognition of its importance, the role of the microbiota in swine resilience has only recently begun to attract focused attention. However, based on the mechanisms described above, it is reasonable to infer that the microbiota, whether through host-mediated regulation or direct action, contributes meaningfully to resilience.

Specifically, the adaptive advantages conferred by the microbiome enhance the host’s buffering capacity, allowing for rapid physiological responses to environmental stressors, pathogen exposure, and dietary fluctuations. In this way, the microbiome extends the host’s functional potential beyond its genome, supplying symbiont-derived traits that help stabilize performance across variable conditions [125].

Currently, microbiability estimates for resilience traits in pigs are modest, at approximately 10% [25]. While this estimate reflects the contribution captured by the model and data used in these studies, it is lower than what might be expected based on biological evidence indicating a central role of the gut microbiota in host–environment interactions and resilience-related processes [126, 127]. This discrepancy may reflect limitations of current study designs and models, such as reliance on single time-point microbiome data, limited taxonomic or functional resolution, and the difficulty of capturing dynamic host–microbiota interactions. Consequently, existing estimates likely represent only a partial contribution of the microbiota to resilience.

Against this backdrop, we propose a review of the current literature on the biological links between the gut microbiota and resilience indicators in pigs. As defined earlier, resilience broadly refers to the ability of animals to maintain certain physiological parameters under stress. In pigs, major stressors include (i) social and behavioral stress, (ii) heat stress, (iii) pathogen exposure, and (iv) disruptions or shifts in the gut microbiota itself. The latter also plays a critical role in nutritional transitions, such as the major dietary change during weaning from milk to solid feed, as well as subsequent feed shifts that occur throughout a pig’s life. The following sections explore how the gut microbiota may influence the pig’s capacity to cope with each of these challenge

### Social stress

In pigs, social stress is a particularly important concern due to their group housing and hierarchical social structure [128]. As in humans and other animals, social stress in pigs is closely associated with neural processes and behavior [129], making it a key factor in physiological resilience. This has important implications not only for animal welfare but also for productivity, as resilient pigs are better able to cope with social stress without negative impact on their welfare and its expression at the phenotypic level. Such phenotypic expression includes behavioral responses and growth-related production traits, notably feeding behavior and body weight [130].

A hypothesized mechanism through which resilience to social stress may be connected by the influence of the gut microbiota on neural function [131]. The connection between the gut microbiota and the central nervous system, commonly referred to as the microbiota–gut–brain axis, is well known in humans [132]. Under this hypothesis, microbial communities and their metabolites can influence neurotransmitter activity, including dopamine signaling, thereby modulating social behavior and aspects of mental well-being [133].

Despite studies showing how fecal microbiota transplantation has been shown to reduce anxiety-like behaviors by altering dopamine levels in the nucleus and modulating dopamine receptor activity in the prefrontal cortex [134, 135]. These dopaminergic changes are crucial, as they are intimately tied to social behavior and emotional regulation. Additionally, research has demonstrated that the microbiota can modulate neurotransmission of aminobutyric acid and glutamate, the primary inhibitory and excitatory neurotransmitters in the central nervous system, respectively. The two neurotransmitters (of aminobutyric acid and glutamate) play essential roles in maintaining neural balance and facilitating numerous physiological processes [136].

Despite a growing body of evidence in humans, empirical evidence linking the gut microbiota to pigs’ resilience to social stress remains scarce. In livestock species such as pigs, support for microbiota–brain interactions is largely based on indirect behavioral and physiological measures, as direct assessment of mental well-being is impossible [137], as the only tool for direct assessment of well-being is the questionnaire. A small number of studies suggest that social and environmental conditions, such as environmental enrichment or social instability, are associated with differences in gut microbiota composition and host health. For example, pigs housed in enriched environments exhibit improved welfare outcomes alongside shifts in bacterial taxa involved in short-chain fatty acid production. Other studies have reported associations between social stress–related behaviors, such as tail biting, and the fecal microbiome. However, these findings are largely correlative, and direct evidence demonstrating a causal role of the gut microbiota in modulating resilience to social stress in pigs is still lacking [138, 139].

### Resistance to pathogens

Microbial resistance is particularly important in intensive swine production systems, where animals are continuously exposed to emerging pathogens while also dealing with persistent, endemic infections [140]. Such resilience is especially critical in the context of infectious diseases like porcine reproductive and respiratory syndrome virus (PRRSV), which remains a major health challenge in the swine industry [141]. The microbiota contributes to pathogen resistance through its complex interactions with (i) the immune system, (ii) the gut–brain axis, as previously discussed, and (iii) the maintenance of blood–brain barrier (BBB) integrity. The gut microbiome plays a central role in shaping both innate and adaptive immune responses. Commensal microbes support immune system development by producing short-chain fatty acids (SCFAs), which influence the function of immune cells, including regulatory T cells that are essential for maintaining immune tolerance [142, 143]. Moreover, the microbiota helps educate the immune system to distinguish between benign antigens and harmful pathogens, promoting oral tolerance and preventing chronic inflammation [144]. Certain bacterial taxa also stimulate the secretion of immunoglobulin A, a key component of mucosal immunity that reinforces gut barrier function and enhances protection against pathogens [145]. Conversely, disruption of microbial balance, dysbiosis, has been associated with immune-mediated disorders such as inflammatory bowel disease, allergies, and autoimmune conditions, highlighting the microbiota’s essential role in immune homeostasis [146]. The microbiota also plays a pivotal role in the gut–brain axis, extending beyond stress regulation as previously mentioned. It influences the production of pro-inflammatory cytokines such as IL-6, TNF-, and IL-1, which contribute to both systemic and neuroinflammation. These cytokines modulate microglial activity, the brain’s resident immune cells, influencing key processes such as synaptic pruning, neuroplasticity, and neural repair [147].

Furthermore, the gut microbiota contributes to the integrity of the BBB, a vital structure for neural protection. Microbial dysbiosis can impair BBB function, allowing peripheral inflammatory mediators to infiltrate the brain, disrupt neural homeostasis, and amplify neuroinflammatory responses [148]. These disruptions are particularly detrimental to resilience, as they may impair behavioral and physiological responses to stress, as mentioned before, ultimately compromising health, disease susceptibility and productivity in swine [149].

### Heat stress

Heat stress is becoming an increasingly serious issue in the swine industry, leading to reduced productivity, reproductive challenges, compromised meat quality, and long-term negative effects on offspring. These consequences highlight the urgent need for effective mitigation and resilience strategies to ensure sustainable swine production. Despite this urgency, most microbial studies on heat stress have focused primarily on documenting changes in gut microbiota composition and microbial metabolites, and their associations with physiological responses under different thermal conditions [150]. In most of these studies, microbiota alterations have been viewed as consequences of heat stress. For example, heat stress has been shown to disrupt gut microbial composition [151], which in turn negatively impacts animal performance [152].

However, the active role of microbiota composition in enhancing resilience to heat stress has been largely underexplored in swine. When considering this active role, a pivotal study by Chevalier et al. [153], demonstrated that the gut microbiota can directly contribute to thermal adaptation. In their experiment, transplanting microbiota from cold-exposed mice into germ-free recipients induced similar cold-resistance traits, highlighting the microbiota’s causal role in driving physiological adaptation. Specifically, the microbiota promoted cold tolerance by remodeling intestinal metabolism, improving nutrient absorption, and stimulating fat browning, mechanisms that collectively enhance energy expenditure during thermal stress. These findings underscore the microbiota’s ability to regulate systemic energy homeostasis in response to environmental challenges [154]. While the original experiment was conducted in mice, similar microbiota-mediated mechanisms have been proposed in other studies [155, 156]. Although research is still needed to confirm these processes in swine, the aforementioned studies allow us to hypothesize that the gut microbiome may play an active role in protecting against heat stress by facilitating metabolic adaptations during thermal exposure. Moreover, we need to report that cold stress can negatively affect pig performance and health, as well. While this can be particularly in young animals, its impact has been largely reduced in modern production systems due to improved housing and climate control. Consequently, recent studies tend to focus on heat stress, although cold stress may still be particularly relevant in specific contexts or production stages.

### Microbiota itself

Although it may seem paradoxical, the resilience of the microbiome is closely linked to the overall health and resilience of the host. In fact, a more resilient gut microbiota often translates into a more resilient organism, including in swine [157]. This is particularly relevant in pigs, which are routinely exposed to a range of microbiota-disrupting factors throughout their lives, including stress, dietary changes, antibiotic treatments, and infections. A stable and robust gut microbial community can either resist such disturbances or recover from them rapidly. Conversely, when the microbiota’s capacity to recover is impaired, it can lead to dysbiosis, an imbalance or disruption of the gut microbial ecosystem. Dysbiosis has been associated with the development of chronic diseases [158]. In general, resilient microbial ecosystems are characterized by high species richness and the presence of functionally important keystone taxa, microorganisms that play a disproportionately large role in shaping the microbial community [22]. These keystone species engage in complex interactions with other microbes, helping to preserve ecosystem stability and function under stress. Furthermore, a resilient microbiota tends to produce a diverse array of beneficial metabolites, such as short-chain fatty acids (SCFAs), which exert immunomodulatory, anti-inflammatory, and barrier-protective effects, thereby supporting host health [159].

In pigs, the protective role of the gut microbiota is particularly evident during periods of nutritional stress, especially around weaning, which represents one of the most critical transitions in early life [159]. The abrupt shift from milk to solid feed is accompanied by marked changes in gut microbial composition and function, often coinciding with increased susceptibility to stress and disease.

Several studies have shown that preserving or supporting the gut microbiome during the weaning period,through interventions such as fecal microbiota transplantation (FMT) or phytogenic feed additives, can enhance microbial diversity, promote beneficial bacterial populations, improve gut morphology, and reduce intestinal inflammation. These findings further highlight the buffering capacity of the gut microbiota, which helps piglets adapt to rapid dietary shifts and mitigates the negative effects of nutritional stress. Collectively, these effects contribute to alleviating weaning-associated stress and improving piglet health and growth performance [160–163].

Taken together, this suggests that resilience could be assessed using longitudinal microbiome data by quantifying microbial profiles at multiple time points and applying “black-box” approaches based on deviations between observed and expected trajectories, analogous to those used for traits such as feed intake or body weight. However, repeated microbiome sampling and sequencing remain costly and logistically demanding, particularly at the scale required for commercial breeding programs. Consequently, despite their conceptual appeal, microbiome-based resilience indicators currently have limited practical applicability.

## Microbiome opportunities to improve resilience

**Fig. 8:**
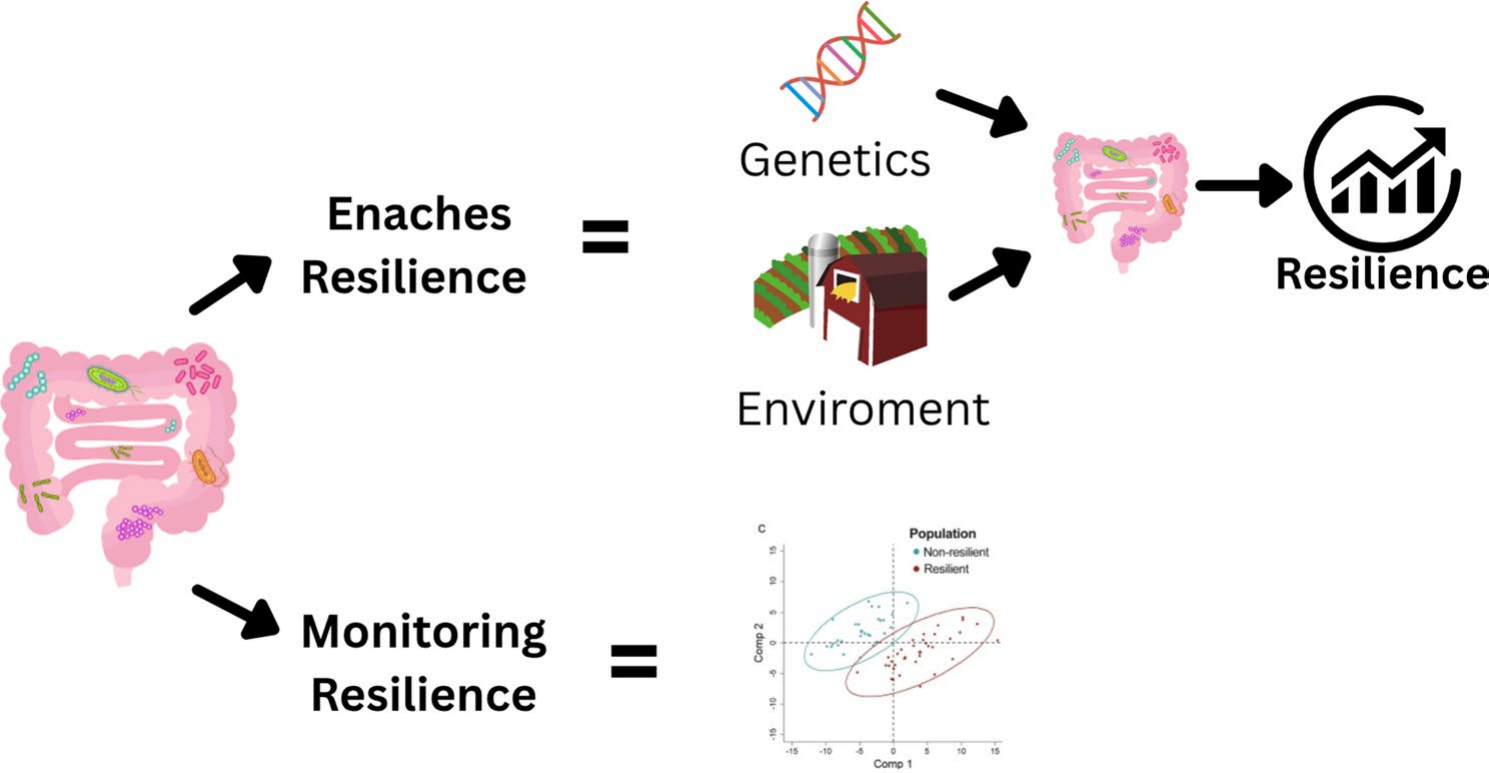
Schematic representation of opportunities to improve resilience. (**i**) Resilience can be actively enhanced, for example, through modulation of the microbiota, which in turn can be influenced by genetic and environmental factors. (ii) alternatively, the microbiota may serve as a biomarker of resilience. In fact, PLS-DA analysis adapted from the study of castro-Rebollo et al. (2023) demonstrated clustering of microbiota profiles associated with animals of higher or lower resilience

As previously discussed, microbiota exhibit potential biological connections with resilience, which may represent valuable opportunities for improving it [164]. Specifically, as illustrated in Fig. 8, resilience may be enhanced via the microbiota in two ways: (i) through targeted manipulation or modulation of the microbial community to actively promote resilience, or (ii) by employing microbiota-related parameters as proxies or biomarkers to facilitate improved monitoring and prediction of resilience.

### Microbiota actively enhances resilience

Microbial communities with a rich alpha diversity and/or promoting beneficial taxa have shown to be associated with an enhanced resilience.

Regarding alpha diversity, as discussed earlier and consistent with Holling et al. [29], systems with greater variability tend to be more resilient. Applying this concept to the microbiome, higher microbial diversity within an animal’s gut or other host-associated ecosystems is often associated with greater adaptability and stability. In this context, alpha diversity, reflecting the richness and evenness of microbial species within a community, is widely recognized as a key indicator of ecosystem stability [165]. For example, in soil ecosystems, increased microbial richness enhances both structural and functional stability, particularly under environmental stresses such as heat [166]. However, this relationship is context-dependent; in some cases, reduced diversity can strengthen microbial networks and maintain ecosystem stability, even if specific functions are compromised [167].

In animals, microbial diversity is increasingly recognized as critical for physiological stability and resilience to stress. In swine, for instance, reduced gut microbial diversity has been linked to decreased resilience [25]. Similar trends appear in other species: amphibians with higher skin microbiome diversity prior to pathogen exposure tend to exhibit higher survival rates [168, 169]. Large-scale studies in wild animals further show that microbial diversity and function correlate closely with host traits such as diet, social behavior, and lifespan, underscoring the adaptive role of a diverse microbiome in responding to environmental challenges [169].

However, this pattern is not universal, Casto-Rebollo et al. [170] found no clear positive association between microbial diversity and resilience. This might suggest that a highly variable microbiome may also reflect buffering capacity or the potential for beneficial microbial community shifts under certain conditions. This supports the idea that resilient animals tend to maintain stable and consistent microbiota compositions under stress, whereas less resilient animals exhibit highly variable and unpredictable microbiome profiles [171]. Such variability may signal a loss of microbial community regulation, often accompanied by reduced diversity and diminished physiological stability.

Similarly, recent research has identified strong associations between specific microbial features, and resilience traits, as mentioned. In swine, lower microbial alpha diversity combined with the presence of certain ASVs and KEGG pathways linked to inflammation negatively correlates with resilience. This suggests that a richer and more balanced microbiota supports physiological stability during stress. For example, partial least squares-discriminant analysis (PLS-DA) has demonstrated that gut microbial composition can reliably distinguish less resilient individuals, highlighting its potential as a biomarker for resilience in livestock management. In rabbits, distinct ASV profiles and taxa such as *Akkermansia* and *Christensenellaceae* R-7 are linked to longevity and resilience, with gut microbiomes showing high accuracy in classifying animals by these traits [24]. Studies in wild lizards reveal this [172], while major taxonomic profiles remain stable across populations, unique ASV signatures and seasonal shifts in families like *Lachnospiraceae* and *Ruminococcaceae* reflect both resilience and plasticity in response to environmental changes. In swine, Mancin et al., 2024 [24] ASVs related to *Mitsuokella*, *Prevotella*, *Oscillospira* and *Ruminococcus* have been associated with beneficial effects.

Additionally, while the use of microbiota data for resilience research is highly promising, several challenges remain in its interpretation. The gut microbiome is a highly complex and dynamic system shaped by multiple factors, including host genetics, environment, diet, and physiological status [173]. Focusing on single amplicon sequence variants (ASVs) or operational taxonomic units (OTUs) may fail to capture the intricate interactions and collective functions within microbial communities [174]. Moreover, phenotypic correlations between individual microbial taxa and resilience-related traits are low or even negative, further limiting their usefulness as robust biomarkers. Simplistic approaches that focus on single taxa risk oversimplify the complexity of gut ecosystems and overlook the synergistic or antagonistic interactions that shape community-level function [175].

### How to improve?

Promoting beneficial microbial species and their connection to resilience is a promising strategy that can be pursued through both genetic selection and management interventions (e.g., dietary modulation).

#### Genetic selection

From a genetic perspective, selecting animals with inherently higher alpha diversity or with microbiota profiles enriched in favorable taxa could potentially improve resilience, as these animals may be better equipped to cope with environmental and physiological stressors. Importantly, studies [19, 176] have shown that certain microbiota traits, including alpha diversity, exhibit moderate to high heritability and relate to genes of the animals. This suggests that they could be viable targets in breeding programs aimed at enhancing the robustness and health of future pig populations. However, to date, genetic selection incorporating microbiome data has not yet been implemented.

Moreover, specific microbial taxa or amplicon sequence variants (ASVs) associated with resilience could theoretically serve as phenotypic markers for indirect selection [177]. For example, selecting for OTUs or ASVs that are both heritable and show positive genetic correlations with resilience traits could provide a means of improving overall robustness in swine.

However, this approach faces significant limitations. As highlighted by Nuñez et al. [176], finding individual ASVs that are simultaneously beneficial for performance and positively correlated with growth has proven challenging. Indirect selection is only effective when the product of heritability and genetic correlation of the microbiota trait exceeds that of the target trait itself. Furthermore, focusing on single microbial features may underestimate the complexity of the microbiome, as microbial taxa often interact within networks, compensating or reinforcing each other’s functions.

To address this complexity, several alternative strategies have been proposed. Saborio-Montero et al. [177] applied principal component analysis to reduce the dimensionality of microbiota data, effectively summarizing complex microbial variation into a few key components that can be linked to resilience traits. Similarly, Weishaar [178] and Christensen et al. [179] developed an approach to collapse microbiota information into a single microbiome-wide effect, providing a simplified metric that can be incorporated into genetic or phenotypic models to enhance predictive power. While these methods help manage the analytical challenges posed by the high dimensionality and intercorrelation of microbial data, they also come with trade-offs, as the biological resolution of individual microbial taxa or functional groups may be lost.

In this context, enterotypes have emerged as a promising strategy to address two major challenges in microbiome-based approaches: capturing the full complexity of the microbial ecosystem while maintaining biological interpretability. The concept of enterotypes was first introduced in 2011 by Arumugam et al. [180] who identified distinct gut microbiome configurations characterized by the dominance of specific microbial taxa. Recent studies in swine have demonstrated that enterotypes are functionally relevant and are associated with multiple production traits, including growth rate, body weight, and average daily gain [181, 182], as well as indicators of health [183]. Furthermore, refined approaches such as entero-signatures, which represent proportional bacterial assemblages rather than fixed categorical classifications, have strengthened the link between enterotypes and host performance [183–185]. Notably, these studies also revealed that enterotype structure is strongly influenced by host genomic background, reinforcing the holobiont concept and highlighting the potential of co-selecting both host and microbiome traits [184].

For practical implementation, breeding companies should consider a phased approach beginning with the establishment of standardized sampling protocols at consistent time points, ideally between 120 and 150 days of age following post-weaning stabilization. Building reference microbiome databases across breeding populations of at least 500 to 1,000 animals per nucleus herd would enable calculation of heritability estimates for microbiome traits and identification of candidate traits showing heritability greater than 0.10 and favorable genetic correlations with resilience indicators. Initial integration should focus on composite traits such as enterotypes or diversity indices rather than individual ASVs, with modest index weights of 5 to 10 given current uncertainties. Validation in commercial environments over 3 to 5 years remains essential before broader implementation.

However, in practice, microbiome-informed selection is currently best viewed as a complementary and exploratory tool, with potential utility at the population level but constrained by biological complexity, limited validation, and the absence of routine implementation in breeding programs.

#### Management practices

An alternative and complementary approach to genetic selection is the active modulation of the microbiota through management practices. The gut microbiome is highly dynamic and responsive to environmental inputs such as diet, housing conditions, and exposure to diverse microbial communities. For example, fecal and pen floor samples typically exhibit greater microbial diversity than oral swabs [185], highlighting the strong influence of the environment on microbial richness and the host’s role as an ecological filter shaping microbiota composition.

Among environmental factors, diet represents a particularly powerful lever for shaping microbiota composition and functionality, with direct implications for animal resilience [186, 187]. During the transition from milk to solid feed, especially when diets are enriched with complex plant-derived fibers, pigs exhibit marked increases in gut microbial diversity [188]. Incorporating diverse dietary fibers and prebiotics promotes the growth of beneficial microbial taxa associated with metabolic flexibility and disease resilience [189]. These microbes often drive the production of short-chain fatty acids, which play key roles in gut integrity and immune modulation. Diets rich in fiber have also been associated with higher abundances of taxa such as Faecalibacterium and Prevotellaceae, which are linked to anti-inflammatory effects and gut homeostasis [190].

Beyond dietary composition, probiotics represent another widely used strategy to modulate the gut microbiota. Probiotic supplementation has been shown to support intestinal microbial balance and enhance immune function in pigs [191]. To be effective, probiotic strains must meet several criteria, including resistance to gastric acidity, the ability to adhere to the intestinal epithelium or mucus layer, and the capacity to modulate host immune and metabolic pathways relevant to resilience [192]. Commonly used probiotic genera in swine production include Lactobacillus, Bifidobacterium, Bacillus, lactic acid bacteria, and yeasts [193]. Both Bacillus subtilis and Saccharomyces cerevisiae have been shown to improve intestinal health by suppressing pathogenic bacteria and supporting beneficial microbial populations, thereby reducing gastrointestinal disorders [194, 195]. Despite their widespread use, the efficacy of probiotics can vary substantially depending on farm-specific conditions, management practices, and environmental context [196].

From a practical perspective, producers may prioritize a limited set of microbiome-oriented interventions based on current evidence and feasibility. High-priority strategies include improving dietary fiber diversity around weaning and implementing gradual dietary transitions to support microbiome stability. Management practices that enhance early maternal microbial transfer, such as ensuring adequate colostrum intake and appropriate timing of cross-fostering, also play an important role. Additional, moderate-priority interventions include the targeted use of probiotics during weaning and other stress periods, provided that selected strains are supported by evidence of gastrointestinal survival and efficacy in pigs. Lower-priority emerging strategies such as fecal microbiota transplantation and targeted next-generation probiotics should currently be considered experimental and reserved for research applications until standardized protocols and regulatory frameworks are established.

In summary, from a practical standpoint, microbiota modulation strategies should prioritize early-life dietary interventions that promote microbial diversity during critical developmental windows, the inclusion of multiple fiber sources to enhance functional redundancy within the gut ecosystem, and the use of well-characterized probiotic strains whose efficacy has been validated under specific production conditions. Importantly, these strategies should be adapted to farm-specific environments rather than applied as universal solutions.

### Microbiota to monitoring resilience

As discussed previously, the microbiota is increasingly recognized as a potential biomarker for monitoring resilience in livestock. Specific operational taxonomic units, amplicon sequence variants, or broader shifts in microbial community composition may act as indicators of animals experiencing physiological stress or serve as early-warning signals for individuals at risk, even before clinical signs become apparent [197]. These microbial markers offer valuable insights into subclinical states that often go undetected by conventional health monitoring systems [198].

However, the routine application of microbiome profiling in animal production is still constrained by practical limitations, most notably cost. For example, even targeted approaches such as 16S rRNA gene sequencing currently cost on the order of approximately 50 USD per sample. The high cost and logistical complexity of repeated sampling and sequencing at the herd or population level currently restrict its feasibility for day-to-day use. While advances in high-throughput sequencing are driving down costs and increasing efficiency, these tools remain more commonly used in research settings or as validation instruments within precision livestock systems, rather than for real-time health surveillance.

Nonetheless, important breakthroughs, particularly in human biomedical research, have begun to inform animal resilience studies. Integrated models that combine microbial and host phenotypic data have significantly improved the identification of potentially causal microbes and enhanced the predictive accuracy of complex traits such as disease resistance and resilience [199, 200]. In the specific context of resilience in livestock, applications of the microbiome as a biomarker are still in their infancy, with only two studies to date exploring this approach. Notably, Mancin et al. (2024) [25] and Casto-Rebollo et al. (2023) [170] have demonstrated that partial least squares discriminant analysis can successfully classify animals as resilient or non-resilient based on their microbial signatures, even before observable phenotypic differences emerge. However, microbiome-based resilience indicators have not yet been validated in independent prospective experiments in which microbiota are first characterized and resilience is subsequently quantified using longitudinal phenotypes.

For practical implementation, microbiome monitoring can provide value in specific contexts through a tiered approach. Research and development applications warrant comprehensive longitudinal sampling with full metagenomic profiling to develop and validate resilience biomarkers. Strategic surveillance through periodic sampling of sentinel animals with targeted 16S sequencing focused on validated biomarker taxa is appropriate for large integrated production systems and high-health multiplier herds, with quarterly sampling of 20 to 50 representative animals recommended. Diagnostic applications through event-triggered sampling following disease outbreaks or performance declines can help identify microbiome-related factors contributing to problems and guide intervention strategies. Key indicators for monitoring include alpha diversity indices where declining diversity may signal stress or dysbiosis, enterotype classification where shifts toward less favorable configurations may precede performance declines, and abundances of beneficial taxa such as Faecalibacterium, Prevotella, and Lactobacillus where declining levels may signal reduced resilience capacity.

To advance microbiome monitoring toward routine application, near-term priorities over the next one to three years should focus on validating existing biomarker candidates in independent prospective studies, developing standardized low-cost sampling protocols, and establishing reference databases for swine populations under different production conditions. Medium-term goals over three to five years should include developing rapid on-farm diagnostic tools such as targeted qPCR panels for key resilience indicators and integrating microbiome data with existing precision livestock farming platforms.

Collectively, these developments mark a shift from descriptive microbiome studies toward a more mechanistic and functional understanding of host-microbe interactions. The emphasis is increasingly on identifying measurable, biologically relevant microbial features that directly influence resilience and could be harnessed in diagnostic tools or targeted interventions. This transition is crucial for advancing microbiome science from research settings into practical applications within precision livestock farming

## Conclusion

Resilience is attracting increasing attention in the swine sector due to its potential to address challenges related to climate change, intensified production systems, and animal welfare. To date, no standardized metrics or universally accepted indicators of resilience have been established. Nevertheless, the gut microbiota—despite initial costs associated with its characterization, emerges as a promising avenue for enhancing resilience. This is largely due to its connection with multiple aspects of resilience, including responses to social stress, heat stress, and disease. Therefore, the microbiota could serve both as a biomarker to identify less resilient animals in advance and as a target for interventions. These could be used to improve resilience through genetic selection or to implement dietary strategies that modify the microbiome. Nevertheless, current research is still limited: to date, only one study in swine, and only two studies when considering all livestock species, have investigated the relationship between the microbiota and resilience. This highlights the need for further studies to explore its full potential as a tool for improving resilience in swine production.

## Data Availability

No datasets were generated or analyzed during the current study.

## Abbreviations

AFS: Automatic feeding systems
VE: Environmental variance
DHGLMs: Double hierarchical generalized linear models
G×E: Genotype by environment interactions
HPTs: Health-performance trajectories
CBC: Complete blood count
PRRSV: Porcine reproductive and respiratory syndrome virus
BBB: Blood-brain barrier
ASVs: Amplicon sequence variants
OTUs: Operational taxonomic units
PLS-DA: Partial least squares discriminant analysis
